# P-MSC-derived extracellular vesicles facilitate diabetic wound healing via miR-145-5p/ CDKN1A-mediated functional improvements of high glucose-induced senescent fibroblasts

**DOI:** 10.1093/burnst/tkad010

**Published:** 2023-10-18

**Authors:** Jianlong Su, Qian Wei, Kui Ma, Yaxi Wang, Wenzhi Hu, Hao Meng, Qiankun Li, Yuehou Zhang, Wenhua Zhang, Haihong Li, Xiaobing Fu, Cuiping Zhang

**Affiliations:** Research Center for Tissue Repair and Regeneration Affiliated to the Medical Innovation Research Division and the 4th Medical Center of Chinese PLA General Hospital, 51 Fucheng Road, Haidian District, Beijing 100048, China; School of Medicine, NanKai University, 94 Weijin Road, Nankai District, Tianjin 300071, China; Research Center for Tissue Repair and Regeneration Affiliated to the Medical Innovation Research Division and the 4th Medical Center of Chinese PLA General Hospital, 51 Fucheng Road, Haidian District, Beijing 100048, China; Research Center for Tissue Repair and Regeneration Affiliated to the Medical Innovation Research Division and the 4th Medical Center of Chinese PLA General Hospital, 51 Fucheng Road, Haidian District, Beijing 100048, China; Research Unit of Trauma Care, Tissue Repair and Regeneration, Chinese Academy of Medical Sciences, 2019RU051, 51 Fucheng Road, Haidian District, Beijing 100048, China; Research Center for Tissue Repair and Regeneration Affiliated to the Medical Innovation Research Division and the 4th Medical Center of Chinese PLA General Hospital, 51 Fucheng Road, Haidian District, Beijing 100048, China; Research Center for Tissue Repair and Regeneration Affiliated to the Medical Innovation Research Division and the 4th Medical Center of Chinese PLA General Hospital, 51 Fucheng Road, Haidian District, Beijing 100048, China; Research Center for Tissue Repair and Regeneration Affiliated to the Medical Innovation Research Division and the 4th Medical Center of Chinese PLA General Hospital, 51 Fucheng Road, Haidian District, Beijing 100048, China; Research Center for Tissue Repair and Regeneration Affiliated to the Medical Innovation Research Division and the 4th Medical Center of Chinese PLA General Hospital, 51 Fucheng Road, Haidian District, Beijing 100048, China; Burn and Plastic Surgery, Zhongda Hospital Affiliated Southeast University, Dingjiaqiao 87, Gulou District, Nanjing 210009, China; Research Center for Tissue Repair and Regeneration Affiliated to the Medical Innovation Research Division and the 4th Medical Center of Chinese PLA General Hospital, 51 Fucheng Road, Haidian District, Beijing 100048, China; Department of Wound Repair, Institute of Wound Repair and Regeneration Medicine, Southern University of Science and Technology Hospital, Southern University of Science and Technology School of Medicine, 6019 Xililiuxian Road, Nanshan District, Shenzhen 518055, China; Research Center for Tissue Repair and Regeneration Affiliated to the Medical Innovation Research Division and the 4th Medical Center of Chinese PLA General Hospital, 51 Fucheng Road, Haidian District, Beijing 100048, China; School of Medicine, NanKai University, 94 Weijin Road, Nankai District, Tianjin 300071, China; Research Unit of Trauma Care, Tissue Repair and Regeneration, Chinese Academy of Medical Sciences, 2019RU051, 51 Fucheng Road, Haidian District, Beijing 100048, China; PLA Key Laboratory of Tissue Repair and Regenerative Medicine and Beijing Key Research Laboratory of Skin Injury, Repair and Regeneration, 51 Fucheng Road, Haidian District, Beijing 100048, China; Research Center for Tissue Repair and Regeneration Affiliated to the Medical Innovation Research Division and the 4th Medical Center of Chinese PLA General Hospital, 51 Fucheng Road, Haidian District, Beijing 100048, China; Research Unit of Trauma Care, Tissue Repair and Regeneration, Chinese Academy of Medical Sciences, 2019RU051, 51 Fucheng Road, Haidian District, Beijing 100048, China; PLA Key Laboratory of Tissue Repair and Regenerative Medicine and Beijing Key Research Laboratory of Skin Injury, Repair and Regeneration, 51 Fucheng Road, Haidian District, Beijing 100048, China

**Keywords:** Diabetic wounds, Mesenchymal stem cells, Extracellular vesicles, miR-145-5p, CDKN1A, Senescence, Fibroblast, Glucose

## Abstract

**Background:**

Persistent hyperglycaemia in diabetes causes functional abnormalities of human dermal fibroblasts (HDFs), partially leading to delayed skin wound healing. Extracellular vesicles (EVs) containing multiple pro-healing microRNAs (miRNAs) have been shown to exert therapeutic effects on diabetic wound healing. The present study aimed to observe the effects of EVs derived from placental mesenchymal stem cells (P-MSC-EVs) on diabetic wound healing and high glucose (HG)-induced senescent fibroblasts and to explore the underlying mechanisms.

**Methods:**

P-MSC-EVs were isolated by differential ultracentrifugation and locally injected into the full-thickness skin wounds of diabetic mice, to observe the beneficial effects on wound healing *in vivo* by measuring wound closure rates and histological analysis. Next, a series of assays were conducted to evaluate the effects of low (2.28 x 10^10^ particles/ml) and high (4.56 x 10^10^ particles/ml) concentrations of P-MSC-EVs on the senescence, proliferation, migration, and apoptosis of HG-induced senescent HDFs *in vitro*. Then, miRNA microarrays and real-time quantitative PCR (RT–qPCR) were carried out to detect the differentially expressed miRNAs in HDFs after EVs treatment. Specific RNA inhibitors, miRNA mimics, and small interfering RNA (siRNA) were used to evaluate the role of a candidate miRNA and its target genes in P-MSC-EV-induced improvements in the function of HG-induced senescent HDFs.

**Results:**

Local injection of P-MSC-EVs into diabetic wounds accelerated wound closure and reduced scar widths, with better-organized collagen deposition and decreased p16INK4a expression. *In vitro*, P-MSC-EVs enhanced the antisenescence, proliferation, migration, and antiapoptotic abilities of HG-induced senescent fibroblasts in a dose-dependent manner. MiR-145-5p was found to be highly enriched in P-MSC-EVs. MiR-145-5p inhibitors effectively attenuated the P-MSC-EV-induced functional improvements of senescent fibroblasts. MiR-145-5p mimics simulated the effects of P-MSC-EVs on functional improvements of fibroblasts by suppressing the expression of cyclin-dependent kinase inhibitor 1A and activating the extracellular signal regulated kinase (Erk)/protein kinase B (Akt) signaling pathway. Furthermore, local application of miR-145-5p agomir mimicked the effects of P-MSC-EVs on wound healing.

**Conclusions:**

These results suggest that P-MSC-EVs accelerate diabetic wound healing by improving the function of senescent fibroblasts through the transfer of miR-145-5p, which targets cyclin-dependent kinase inhibitor 1A to activate the Erk/Akt signaling pathway. P-MSC-EVs are promising therapeutic candidates for diabetic wound treatment.

## Graphical Abstract

**Graphical Abstract:**
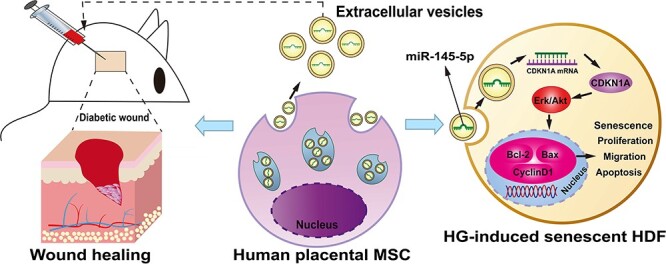

Highlights

- This study is the first to report that miRNA-145-5p was highly enriched in P-MSC-EVs.
- This study is the first to show that P-MSC-EVs improved the cell function of HG-induced senescent HDFs by transferring miR-145-5p.
- This study is the first to prove that miR-145-5p could enhance the function of HG-induced HDFs by targeting CDKN1A.

## Background

Diabetes mellitus is a chronic and multifaceted metabolic disease characterized by continuously and rapidly increasing morbidity, especially in the elderly population. Nearly 20% of diabetic patients worldwide develop delayed or nonhealing chronic wounds. In China, diabetes has also become the major cause of chronic wounds in hospitalized patients [1–4]. Leg and foot ulcers are the most common chronic wounds in diabetic patients. If not properly diagnosed and treated, diabetic foot ulceration results in amputation in 15–27% of patients [5]. Conventional treatments [6], including skin substitutes, debridement and pressure offloading, are sometimes ineffective and fail to provide optimum clinical outcomes for diabetic foot ulcers, which increases the risk of limb amputation.

Human dermal fibroblasts (HDFs) are involved in and responsible for normal wound healing. They can migrate to the wound bed to proliferate and differentiate into myofibroblasts, providing a scaffold for repopulating cells through synthesizing and secreting extracellular matrix, as well as expressing cytokines and growth factors, thereby promoting wound closure. However, substantial evidence suggests that a high-glucose (HG) microenvironment impairs the proliferation, migration, and antiapoptotic abilities of fibroblasts and inhibits fibroblast differentiation into myofibroblasts, as well as extracellular matrix production, thus blocking wound repair [7]. Therefore, improving the functional state of fibroblasts in a HG environment is crucial to promoting diabetic wound healing.

Increasing lines of evidence indicate that mesenchymal stem cells (MSCs) are beneficial for regenerative medicine due to their self-renewal and multilineage differentiation abilities. However, many studies have proven that the benefits of MSC-based cell transplantation are mainly attributed to paracrine products rather than direct differentiation. Extracellular vesicles (EVs), which are paracrine factors released from MSCs, have been demonstrated to possess almost equivalent biological effects to their parent cells [8], representing a prospective stem cell-free therapeutic option [9]. EVs derived from MSCs with different tissue origins have been reported to accelerate diabetic wound healing. For example, EVs derived from bone marrow MSCs promote the proliferation and migration of chronic wound fibroblasts and endothelial cells [10]. EVs derived from induced pluripotent stem cells tend to enhance collagen maturity and re-epithelization [11]. EVs derived from placental MSCs (P-MSC-EVs) have great potential in promoting the proliferation and migration of endothelial cells and reducing scar formation [12,13]. However, the effects of P-MSC-EVs on chronic diabetic healing and the function of senescent HDFs need to be confirmed, and the potential mechanisms also need to be elucidated.

EVs contain a variety of endogenous biological cargos, such as proteins, lipids and microRNAs (miRNAs), involved in mediating intercellular crosstalk. miRNAs are highly conserved noncoding small RNAs (18–24 nucleotides) that can bind to the 3′-untranslated regions (3′-UTRs) of mRNAs to cause mRNA degradation or translation inhibition. Further study demonstrated that miRNAs in EVs have a crucial regulatory roles in wound healing [14]. For example, miR-19b in EVs derived from human adipose-derived stem cells promoted fibroblast proliferation and migration by targeting CC chemokine ligand 1 and regulating the transforming growth factor-β (TGF-β) pathway, which in turn accelerated skin wound healing [15]. EVs derived from human adipose-derived stem cells transferred miRNA-125a to endothelial cells and promoted angiogenesis in wounds byinhibiting Delta-like 4 expression.

MiR-145-5p has been widely studied and is reported to participate in cellular proliferation, migration, apoptosis, differentiation, metastasis and immunoregulation [16–18], which makes it an attractive target for cancer therapy. A growing number of studies have noted that miR-145-5p expression is highly associated with diabetes and HG -induced injury [19–25], although conflicting results have been reported when investigating the relationship between miR-145-5p expression and the hyperglycemic conditions [20,23–25]. Furthermore, preliminary work has shown that miR-145-5p is involved in cell senescence, and the overexpression of miR-145-5p was found to be able to rejuvenate the phenotype and augment the functionality of adipose stem cells derived from old subjects [26,27]. Recently, miR-145-5p has been identified in EVs from tissue, plasma, urine and cells [19,28–31]. However, the biological role of exosomal miR-145 in HG-induced HDFs and diabetic wound healing is unclear.

In the present study, we observed the effects of P-MSC-EVs on diabetic wound healing and collagen deposition in wounds. P-MSC-EVs were used to treat HG-induced senescent fibroblasts and the antisenescence, proliferation, migration and antiapoptotic abilities of fibroblasts were examined. Next, the candidate miRNAs responsible for the function of P-MSC-EVs were confirmed *in vitro* and *in vivo*. Furthermore, the target genes of candidate miRNAs were identified by RNAi experiments. Finally, we found that P-MSC-EVs accelerated diabetic wound healing by improving the function of senescent fibroblasts through the transfer of miR-145-5p, which targets cyclin-dependent kinase inhibitor 1A (CDKN1A) to activate the extracellular signal regulated kinase (Erk)/protein kinase B (Akt) signaling pathway.

## Methods

### Cell culture

P-MSCs isolated from placenta were obtained from our laboratory and cultured in Dulbecco’s modified Eagle’s medium/F12 (Gibco, USA) containing 10% EVs-free fetal bovine serum (FBS; Gibco, USA) and 1% penicillin and streptomycin (Gibco, USA) [32]. Cells were cultured at 37°C with 5% CO_2_ in a humidified environment, and cells at passages 3–6 were employed for isolation of EVs. HDFs were isolated using previously described protocols [33] and cultured at 37°C with 5% CO_2_ in a humidified environment. For HG-induced experiments, HDFs at passage 6 were cultured in Dulbecco’s modified Eagle’s medium with glucose at final concentrations of 5.5 and 35 mM [32] for 10 days during which the medium was changed every 72 h. After the desired time, cell senescence was assessed by senescence-associated beta-galactosidase (SA-β-gal) staining, cell proliferation was evaluated by EdU (5-ethynyl-2'-deoxyuridine) and cell counting kit-8 (CCK-8) assay, cell cycle and cell apoptosis were analyzed by flow cytometry, and cell migration was observed by scratch and transwell assays.

### Isolation and identification of P-MSC-EVs

EVs were harvested according to previously described methods [34]. Briefly, the cell culture medium was collected and centrifuged at 2000 x g for 10 min to remove cellular debris. The supernatants were then collected and centrifuged at 10,000 x g for 30 min, and the new supernatants were collected and ultracentrifuged at 100,000 x g for 75 min. Next the deposit was obtained and resuspended with 1 ml phosphate-buffered saline (PBS) and the resuspension was ultracentrifuged again at 100,000 x g for 75 min after filtering with a 0.22 μm filter (Steritop™ Millipore, MA, USA). The new deposit (P-MSC-EVs) was resuspended with PBS and stored at −80°C. All the centrifugation steps were conducted at 4°C.

In addition, for the identification of P-MSC-EVs, a transmission electron microscope (HITACHI HT7700, Hitachi High-Technologies, Japan) was used to capture the morphology, a Zetaview instrument (Particle Metrix, Meerbusch, Germany) was used to measure the density and size, and western blotting was used to examine the expression of CD9, tumor susceptibility gene101 (TSG101), and calnexin in the cell lysis, EVs-depleted medium and P-MSC-EVs.

### *In vivo* administration of P-MSC-EVs

Eight-week-old male diabetic mice (BKS-Dock Leprem2Cd479, db/db, weighing 26–30 g) were purchased from The Center for Experimental Animals, Jicuikang Company. All procedures followed the guidelines of the Animal Research Committee of the Chinese PLA General Hospital. After the mice were shaved and anesthetized, full-thickness excisional skin wounds on the dorsum (10 mm in diameter) were created. All animals were randomized into control (PBS) and P-MSC-EVs groups (n = 5). P-MSC-EVs were labeled with PKH26 (Sigma-Aldrich, Germany) according to the manufacturer’s protocol. Observation of the fluorescent image was carried out using a Bruker in vivo imaging system Fx Pro two days after injection. P-MSC-EVs (100 μl; 4.56 x 10^10^ particles/ml) were injected around the wounds at four injection sites (25 μl per site) every two days for 14 consecutive days. The wounds of each group were photographed on Days 0, 4, 8, 12, and 16 after surgery and the wound closure rate was measured using Image-Pro Plus 6 software (Media Cybernetics, Bethesda, USA) and calculated using the equation: wound closure rate (%) = [(initial wound area − actual wound area at day *x*)/initial wound area] × 100%.

### Histological analysis

After different treatments, the mice were sacrificed at the projected time points (Days 4, 8, 12 and 16) and skin specimens were obtained and fixed in 4% paraformaldehyde postoperatively. Then, tissues were dehydrated using graded ethanol, embedded in paraffin and subsequently cut into 4-μm-thick sections, followed by hematoxylin and eosin staining (Solarbio, Beijing, China) and Masson’s trichrome staining (Solarbio, Beijing, China) according to the manufacturer’s instructions. Scar width and the degree of collagen maturity or collagen volume fraction were evaluated according to the literature as previously described [35,36]. Images of stained sections were captured using a digital imaging scanning system (Precipoint M8; Precipoint, Freising, Germany) and the results were analyzed using Image-Pro Plus 6 software.

### Immunofluorescence staining

Immunofluorescent staining was performed as described elsewhere [37]. Briefly, paraffin-embedded slides of skin samples obtained on Day 16 post-wounding were processed with 1 h heating at 60°C, deparaffinization in xylene, rehydration in graded ethanol, 15 min antigen retrieval in citrate buffer, 15 min cell permeation using 0.3% Triton x-100 and 2 h of unspecific antigen blocking using 10% goat serum, and then were incubated with primary antibodies against p16INK4a (1 : 50; sc-1661, Santa Cruz Biotechnology) at 4°C overnight. The next day, the slides were washed three times with PBS and incubated with Alexa Fluor 647 fluorescence secondary antibody (1 : 200, Invitrogen) for 1 h in dark at room temperature. Next, the slides were stained with DAPI (4',6-diamidino-2-phenylindole) and mounted. Finally, immunofluorescence images were recorded using confocal microscope (Leica, Germany).

### SA-β-gal staining

SA-β-gal staining was used to evaluate SA-β-gal expression in HG-induced HDFs and was carried out according to the manufacturer’s instructions of the SA-β-gal staining kit (Sigma-Aldrich, Germany). Briefly, HDFs were washed and fixed with 4% paraformaldehyde for 20 min and then incubated with the SA-β-gal staining solution overnight at 37°C under CO_2_-free conditions. Subsequently, HDFs were observed under a phase-contrast microscope (Leica DMI 3000B, Solms, Germany). The proportion of SA-β-gal-positive cells was measured by counting the blue cells *vs* total cells.

### Uptake assay of P-MSC-EVs

For the EV uptake assay, 4 μg PKH67 (Sigma-Aldrich, Germany) was used to label 50 μg of P-MSC-EVs at room temperature for 5 min. Then, the EVs were washed with PBS and recollected via ultracentrifugation to remove non-solubilized material, followed by sterilization through a 0.22 μm membrane filter. HDFs were co-incubated in 24-well plates with PKH67-labeled EVs. The internalization of P-MSC-EVs by HDFs was counterstained with phalloidin-rhodamine B (cytoskeleton) and DAPI (cell nucleus) and observed under confocal microscope (Leica, Germany). The HG-induced HDFs were treated with P-MSC-EVs at different concentrations (2.28 × 10^10^ particles/ml, 4.56 × 10^10^ particles/ml) for the cell proliferation assay, cell cycle, scratch assay, transwell assay, and apoptosis assay. At the same time, the expression of cyclinD1, Bcl-2 and Bax was also analyzed by real-time quantitative PCR (RT-qPCR) and western blotting.

### Detection of miRNAs in P-MSC-EVs and target gene prediction

MiRNAs in P-MSC-EVs were isolated and the expression of miRNAs, including miR-602, miR-1290, miR-25-3p, miR-23a-3p, miR-23b-3p, miR-3187-3p and miR-145-5p, in EVs was confirmed by RT-qPCR. The results demonstrated that miR-145-5p is the most abundant miRNA in EVs. Next, miR-145-5p targets including CDKN1A and calcium/calmodulin dependent protein kinase 1D (CAMK1D) were predicted by using the following online software: TargetScan (TargetScanHuman 8.0) [38], miRWalk [Home-miRWalk (uni-heidelberg.de)], miRDB (miRDB-MicroRNA Target Prediction Database), starBase [ENCORI: The Encyclopedia of RNA Interactomes. (sysu.edu.cn)], miRTarBase [miRTarBase: the experimentally validated microRNA-target interactions database (cuhk.edu.cn)] and DIANA Tarbase [(DIANA tools—Tarbase v8 (uth.gr)] [39,40].

### MiRNA interference

We obtained miR-145-5p mimics, mimic negative control (miR-NC), miR-145-5p inhibitors, inhibitor negative control (inhibitor-NC), three small interfering RNAs (siRNAs) targeting CDKN1A (si-CDKN1A #1, 2 and 3), three siRNAs targeting CAMK1D (si-CAMK1D #1, 2 and 3), and the universal negative control siRNA (si-NC) from GenePharma (Shanghai, China). The detailed sequences are shown in (Table S1, see online supplementary material). MiR-145-5p mimics, si-CDKN1A and si-CAMK1D were labeled with cyanine 3 dyes (Cy3, red). The transfection efficiency was assessed by flow cytometry.

To confirm that miR-145-5p inhibitors can block the effect of miR-145-5p, miR-145-5p inhibitors (100 nM) or inhibitor-NC (100 nM) were transfected into HG-induced HDFs pre-incubated with P-MSC-EVs (4.56 × 10^10^ particles/ml) with the aid of Lipofectamine 3000 (Invitrogen, MA, USA). To confirm that miR-145-5p mimics can simulate the effect of P-MSC-EVs, miR-145-5p mimics or miR-NC were transfected into HG-induced HDFs with the aid of Lipofectamine 3000. The expression of miR-145-5p was detected by RT-qPCR. We also performed a cell proliferation assay, cell cycle, scratch assay, transwell assay, and apoptosis assay. Simultaneously, the expression of cyclinD1, Bcl-2 and Bax were analyzed by RT-qPCR and western blotting.

To further identify the target genes of miR-145-5p, the expression of CDKN1A and CAMK1D were detected by RT-qPCR after transfection with miR-145-5p mimics. Next, si-CDKN1A (#1, 2 and 3, 200 nM), si-CAMK1D (#1, 2 and 3, 200 nM) and si-NC (200 nM) were transfected into HG-induced HDFs to find the most effective si-CDKN1A and si-CAMK1D. Finally, cell proliferation, cell migration, cell cycle, cell apoptosis, and the expression of Akt, phosphorylated Akt (p-Akt), Erk1/2 and p-Erk1/2 were detected after transfecting the effective si-CDKN1A or si-CAMK1D.

### Cell proliferation assay

CCK-8 and EdU assays were conducted to evaluate the proliferative capacity of HDFs. For the CCK-8 assay, HDFs were seeded into 96-well plates at a density of 2 × 10^3^ cells/well (four replicates per group) and cultured in medium supplemented with or without P-MSC-EVs, miRNA and siRNA. At a planned time point, CCK-8 reagent was added to the cells in serum-free medium and incubated for 3 h, followed by measurement of absorbance at 450 nm. For the EdU assay, 1 × 10^4^ HDFs/well were added to 24-well plates and EdU (Beyotime, Shanghai, China) staining was carried out 48 h after P-MSC-EVs, miRNA or siRNA treatment according to the provided protocols. Then the results were visualized by a fluorescence microscope.

### Cell migration assay

The migration of HDFs was evaluated by means of scratch and transwell assays. For the scratch assay, 2 × 10^5^ HDFs/well were plated into a 12-well plate (three replicates per group). When 90% confluence was reached, cells were scratched with a 1-ml pipette tip and then gently washed with PBS to remove floating cells. After different treatment as described above, the cells were photographed at 0, 24 or 32 h post-scratch and measured by Image-Pro Plus 6.0 software. The migration area was calculated as (%) = [(original gap area − gap area at *x* h)/original gap area] × 100%. For the transwell assay, 24-well transwell inserts (Corning, NY, USA) were used with 8-μm-pore-sized filters. HDFs (1 × 10^4^ cells/well) were suspended in 100 μl of low-serum medium (containing 5% FBS), and then plated into the upper chamber filled with 600 μl of HG medium (containing 10% FBS) supplemented with or without P-MSC-EVs, miRNA, and siRNA. After 24 h incubation, cotton swabs were used to scrape off cells attached to the upper surface of the filter membrane. The cells attaching on the lower surface were then stained using 1% crystal violet (Solarbio, Beijing, China) for several minutes at room temperature. Finally, the migrated cells were photographed and counted under an optical microscope.

### Flow cytometry

Cell cycle and cell apoptosis assays were performed by flow cytometry. For the cell cycle assay, HDFs (8 × 10^5^ cells/well) were seeded in six-well plates and cultured by different treatments for 48 h. Then, the cells were collected and fixed in 70% cold ethanol at 4°C overnight, followed by resuspension with DNase-free RNase at 37°C for 30 min and subsequent addition of propidium iodide (PI) for DNA staining (30 min, 4°C). PI fluorescence was examined with a flow cytometer (BD FACS Calibur™, BectonDickinson, NJ, USA).

For the cell apoptosis assay, HDFs that had received different treatments for 48 h, as described above, were treated with carbonyl cyanide *m*-chlorophenyl hydrazine (10 μM; Solarbio, Beijing, China) in an incubator for 20 min to induce apoptosis. The cells were then collected and washed with ice-cold PBS and resuspended with the buffer provided in the cell apoptosis kit (Solarbio, Beijing, China). Subsequently, 100 μl of cell suspension, 200 μl of PBS, 5 μl of Annexin V-FITC (Fluorescein Isothiocyanate) reagent and 5 μl of PI regent were sequentially mixed and incubated for 15 min at room temperature in the dark. Cell apoptosis was then immediately detected via flow cytometry.

### RT-qPCR

Total RNA was extracted from cells and P-MSC-EVs using an miRNeasy Mini Kit (QIAGEN, Hilden, Germany) according to the manufacturer’s instructions. For mRNA detection, cDNA was synthesized using a FastKing gDNA Dispelling RT Super Mix kit (Tiangen, Beijing, China), followed by qPCR reactions using a SYBR Green Super-Real PreMix Plus kit (Tiangen, Beijing, China), which was performed on an ABI Real-Time PCR Detection System (ABI7500 FAST, Thermo Fisher Scientific, MA, USA). For determination of miRNA expression in P-MSC-EVs, a synthetic analog of non-human cel-miR-39 (QIAGEN, Hilden, Germany) was spiked in 10 μl of a 5-fmol/μl stock to normalize RNA extraction efficiency. Then, cDNA synthesis and subsequent qPCR reactions were conducted using a Hairpin-itTM microRNA and U6 snRNA Normalization RT-PCR Quantitation Kit (GenePharma, Shanghai, China) according to the manufacturer’s protocol. For determination of miRNA expression in cells, cDNA synthesis of miRNAs and qPCR reactions were performed as described above. The mRNA-specific forward and reverse primers as well as miRNA-specific forward primers and the universal reverse primer were designed and synthesized by Sangon Biotech (Shanghai, China). The primer sequences are listed in Tables S2 and S3 (see online supplementary material), respectively. The relative mRNA and miRNA expression levels were normalized to β-actin and U6 levels,, respectively, and were quantified according to the 2^−ΔΔ^CT method.

### Western blotting

Total proteins were extracted from fibroblasts and EVs as previously described [34]. A total of 20 μg of protein extract aliquots were loaded onto sodium dodecyl sulfate–polyacrylamide gel electrophoresis and then electro-transferred onto polyvinylidene fluoride membranes (Millipore, Billerica, MA, USA). Then the polyvinylidene fluoride membranes were blocked with 5% skim milk at room temperature for 2 h to block nonspecific binding. Subsequently, the membranes were probed with specific primary antibodies at 4°C overnight, followed by incubation with horseradish peroxidase-conjugated secondary antibodies at room temperature for 1 h. Finally, the immunoreactive bands were visualized using enhanced chemiluminescence reagent (Thermo Fisher Scientific, Waltham, USA) and imaged by a ChemiDoc XRS Plus luminescent image analyzer (Bio-Rad). Protein band intensity from three independent experiments was measured by Image-Pro Plus 6.0 software and β-actin was used for normalization. Antibodies used in the study were as follows: CD9 (Abcam, #ab92726, 1 : 1000), TSG101 (#ab30871, 1 : 1000), calnexin (#ab2259, 1 : 1000), cyclin D1 (#ab16663, 1 : 1000), Bax (#ab32503, 1 : 1500) and Bcl-2 (#ab185002, 1 : 1000) and were obtained from Abcam (Cambridge, UK); anti-phosphorylate Akt (#9271, 1 : 1000), anti-Akt (#9272, 1 : 1000), anti-p-Erk1/2 (#4370, 1 : 1000), anti-Erk1/2 (#4695, 1 : 1000) and anti-β-actin (#3700, 1 : 5000) were obtained from Cell Signaling Technology (Danvers, MA, USA); and horseradish peroxidase conjugated anti-rabbit IgG (#zb-2301, 1 : 2500) and anti-mouse IgG (#zb-2305, 1 : 2500) were obtained from (ZhongShanjinQiao, Beijing, China).

### Application of miR-145-5p *in vivo*

Animal experiment with mice was also performed to evaluate the potential role of miR-145-5p in P-MSC-EVs. Full-thickness excisional skin wounds in diabetic mice were created as described above. PBS (100 μl), miR-145-5p agomir (agomiR-145-5p) (20 OD (optical density)/ml, 100 μl, GenePharma, Shanghai, China) or miR-145-5p antagomir (antagomiR-145-5p) (10 OD/ml, 100 μl, GenePharma, Shanghai, China) was injected around the wounds at four sites (25 μl per site) every two days for 14 consecutive days (n = 5). Wound closure and histological analyses were performed by the methods described in the aforementioned animal experiment. Additionally, an *in vivo* fluorescent image of agomiR-145-5p labeled with Cy3 was taken by Bruker in vivo imaging system Fx Pro on Day 2 after injection. The sequences of agomiR-145-5p and antagomiR-145-5p are shown in Table S1, see online supplementary material.

### Statistical analysis

All quantitative data are presented as the mean ± SD. Student’s *t-*test was used to analyze significant differences between two groups. One-way ANOVA (Analysis of Variance) with the Dunnett *post hoc* test was adopted to compare differences of more than two groups at the same point and two-way ANOVA with the Dunnett *post hoc* test was performed to analyze differences between multiple groups at different time points. All statistical analyses were conducted using GraphPad Prism 7.0 software. Differences were considered statistically significant at a level of ^*^p < 0.05, ^*^^*^p < 0.01, ^*^^*^^*^p < 0.001 and ^*^^*^^*^^*^p < 0.0001.

## Results

### Characterization of P-MSC-EVs

**Figure 1:**
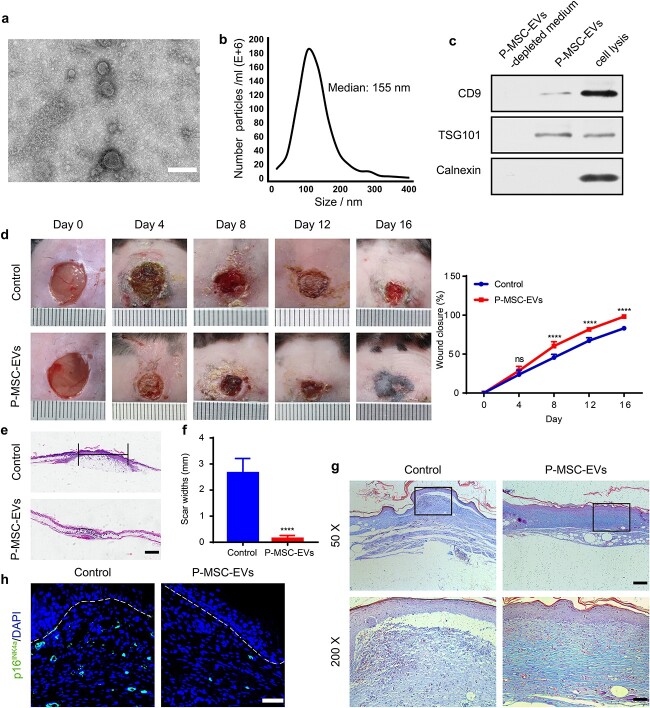
P-MSC-EVs promoted cutaneous wound healing in diabetic mice**.** (**a**) Morphology of P-MSC-EVs observed with transmission electron microscopy. Scale bar: 100 nm. (**b**) Particle size distribution of P-MSC-EVs measured by NTA. (**c**) Expression levels of CD9, TSG101 and Calnexin. (**d**) General view and the rate of wound closure with different treatments on Days 0, 4, 8, 12, and 16 after wounding, n = 5 per group. (**e**, **f**) H&E staining of wound sections treated with PBS and P-MSC-EVs on Day 16 after the operation. Scale bar: 1 mm. (**g**) Masson’s trichrome staining on Day 16 post-wounding. Collagen fiber is stained in blue. Scale bars: 2 mm (upper), 500 μm (lower). (**h**) Expression of p16INK4a in fibroblasts *in vivo* after treatment with P-MSC-EVs. Scale bar: 50 μm. Compared with the control group, ^*^^*^^*^^*^p < 0.0001; *ns* no significance. *P-MSC-EVs* extracellular vesicles derived from human placental mesenchymal stem cells, *NTA* nanoparticle tracking analysis, *H&E* hematoxylin and eosin, *PBS* phosphate-buffered salin, *TSG101* tumor susceptibility gene 101

The conditioned medium of P-MSCs was collected for EV isolation. The characteristics of P-MSC-EVs were evaluated by transmission electron microscopy (TEM), nanoparticle tracking analysis (NTA), and western blotting. The ultrastructure of P-MSC-EVs was revealed by TEM and is presented in Figure 1a and is cup shaped with a diameter of ~100 nm. NTA showed that the size of P-MSC-EVs basically ranged from 30 to 200 nm, and the average particle size was 115.5 nm (Figure 1b), which is consistent with the previously reported size distribution of EVs [41]. Representative markers of EVs including CD9 and TSG101, were expressed by P-MSC-EVs. Calnexin, which is a negative protein marker of EVs, was not detectable in our study (Figure 1c). These results demonstrate that the characteristics of the isolated nanoparticles from P-MSCs matched the criteria of EVs.

### P-MSC-EVs promoted cutaneous wound healing in diabetic mice

We next explored the ability of P-MSC-EVs to promote diabetic wound healing by creating full-thickness cutaneous wounds on the dorsal skin of diabetic mice. *In vivo* image analysis confirmed the continuous retention of P-MSC-EVs two days after injection (Figure S1, see online supplementary material). In addition, as shown in Figure 1d, gross observations indicated significantly accelerated wound closure rates in the P-MSC-EV group on Days 8, 12, and 16 after wounding in comparison with those in the PBS group. Particularly, on Day 16 after wounding, the P-MSC-EVs-treated wounds were almost closed while ~20% of the area of the control wounds remained. Similarly, the two groups had significant differences in scar widths on Day 16 post-wounding (P-MSC-EV group: 0.13 ± 0.05 mm; PBS group: 2.6 ± 0.25 mm, *p* < 0.0001) (Figure 1e, f). Moreover, P-MSC-EV-treated wounds were characterized by larger amounts of wavy collagen fibers and better-organized collagen deposition than the control wounds (Figure 1g). In addition, immunofluorescence staining showed a large number of p16INK4a-positive HDFs in the wound beds in the PBS group, while p16INK4a-positive HDFs were barely found in the P-MSC-EV group (Figure 1h). These data indicate that P-MSC-EV treatment improved collagen deposition and HDF senescence and accelerated diabetic wound healing.

### HG induced senescence and impaired the cell function of HDFs

**Figure 2:**
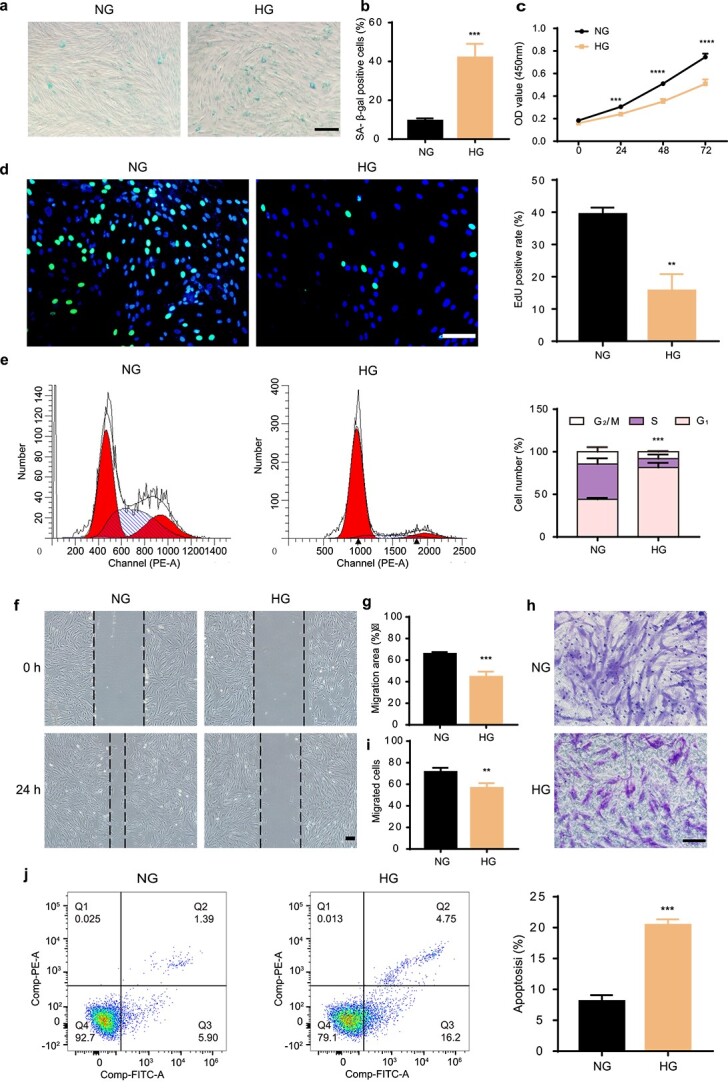
HG induced the senescence and impaired the function of HDFs. (**a**) SA-β-gal assay was performed with SA-β-gal-kit. Scale bar: 200 μm. n = 3 per group. (**b**) Statistical analysis of SA-β-gal positive cells in different treatment groups. (**c**, **d**) Cell proliferation rate was measured by CCK-8 and EdU incorporation assays (green: EdU staining; blue: Hoechst staining). Scale bar: 100 μm. n = 4 per group. (**e, j**) Cell cycle distribution and rates of apoptosis detected by flow cytometry. n = 3 per group. (**f**–**i**) HDF migration ability was determined by scratch assay (scale bar: 200 μm) and transwell assay (scale bar: 100 μm). n = 4 per group. Compared with the NG group, ^*^^*^p < 0.01; ^*^^*^^*^p < 0.001; ^*^^*^^*^^*^p < 0.0001. *HDFs* human dermal fibroblasts, *HG* high glucose, *CCK-8* cell counting kit-8, *NG* normal glucose

To simulate a HG microenvironment *in vitro*, HDFs were cultured with glucose at a final concentration of 35 mM as previously reported [34,42]. After 10 days of HG treatment, the expression of SA-β-gal was increased in the HG group compared with the normal glucose (NG) group (Figure 2a, b), which is consistent with the previously published reports [42]. Next, we observed the effects of HG on the proliferation and migration of HDFs. The results of the CCK-8 assay and EdU incorporation assay showed lower proliferation in the HG group (Figure 2c, d). The result was further confirmed using a cell cycle assay. The results revealed that the HG group exhibited lower proportions of S and G_2_/M subpopulations, as well as higher proportions of G_0_/G_1_ subpopulation than the NG group (Figure 2e, *p* < 0.05). The migration of HDFs was evaluated with wound scratch and transwell assays. The scratch assays demonstrated that the migration rates in the HG group were decreased significantly compared with those in the NG group (Figure 2f, g). Consistent with thisfinding, the number of migrated HG-treated HDFs was less than the number of migrated NG-treated HDFs in transwell assays (Figure 2h, i). Additionally, a correspondingly increased cell apoptosis rate was observed in the HG group (Figure 2j, *p* < 0.001). Together these results suggest that glucose at a concentration of 35 mM induced senescence and impaired the function of HDFs.

### P-MSC-EVs improved the function of HG-induced senescent HDFs

**Figure 3:**
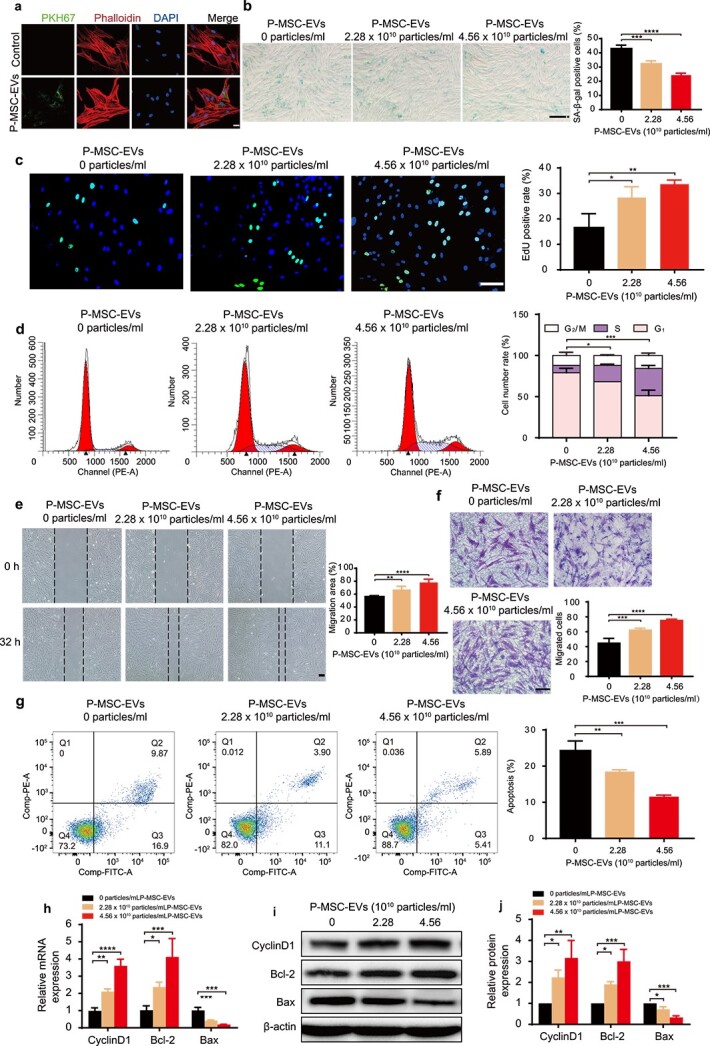
P-MSC-EVs improved the functions of HG-induced senescent HDFs. (**a**) PKH67-labeled P-MSC-EVs were taken up by HG-induced senescent HDFs as indicated by a green fluorescence signal. Cytoskeleton is marked by red fluorescence (phalloidin). Scale bar: 25 μm. (**b**) SA-β-gal expression in HDFs was detected after treatment with P-MSC-EVs. Scale bar: 200 μm. n = 3 per group. (**c**) Effect of P-MSC-EVs on the proliferation of HG-induced senescent HDFs was measured by EdU incorporation assay (green: EdU staining; blue: Hoechst staining). Scale bar: 100 μm. n = 4 per group. (**d**, **g**) Cell cycle distribution and rates of apoptosis detected by flow cytometry. n = 3 per group. (**e, f**) Migration ability of HG-induced senescent HDFs was determined using scratch assay (scale bar: 200 μm) and transwell assay (scale bar: 100 μm). n = 4 per group. (**h**–**j**) Effects of P-MSC-EVs on the expression of cyclin D1, Bcl-2 and Bax in HG-induced senescent HDFs were assessed by RT-qPCR and western blotting. n = 3 per group. Compared with the control group, ^*^p < 0.05; ^*^^*^p < 0.01; ^*^^*^^*^p < 0.001; ^*^^*^^*^^*^p < 0.0001. *P-MSC-EVs* extracellular vesicles derived from human placental mesenchymal stem cells, *HDFs* human dermal fibroblasts, *HG* high glucose

To illustrate the multiple effects of P-MSC-EVs on HDFs, we first determined whether P-MSC-EVs could be internalized by HDFs. P-MSC-EVs were labeled with PKH67 and then incubated with HDFs for 12 h. After fixation, recipient cells were stained with phalloidin. As shown in (Figure 3a), all HDFs were stained green after incubation with labeled P-MSC-EVs, demonstrating that PKH67-tagged P-MSC-EVs had been transferred to the cells and were mainly localized around the perinuclear region in HDFs. Next, P-MSC-EVs at different concentrations were added to the culture medium of HG-induced HDFs. Figure 3b shows that the SA-β-gal expression in HDFs was decreased in the P-MSC-EV group. The results of EdU incorporation and CCK-8 assay indicated that treatment with 2.28–4.56 × 10^10^ particles/ml P-MSC-EVs enhanced the proliferation of HG-induced HDFs in a dose-dependent manner (Figure 3c and Figure S2, see online supplementary material), which was confirmed by a cell cycle assay showing that more cells were in the S and G_2_/M phases than HDFs treated with HG alone (Figure 3d). The results of the scratch assay indicated that the migration of HG-induced HDFs was improved after P-MSC-EV treatment (Figure 3e, *p* < 0.01). In accordance with this finding, many more migrated HDFs were observed in the P-MSC-EV-treated group (Figure 3f, *p* < 0.0001). Additionally, P-MSC-EVs were capable of lowering the apoptotic rate of HG-induced HDFs (Figure 3g). Consistent with these results, we observed elevated expression of proliferation-related protein cyclin D1, upregulated anti-apoptotic Bcl-2 expression, and decreased pro-apoptotic Bax expression at the mRNA and protein levels after P-MSC-EV treatment (Figure 3h–j). Together, these findings indicate that P-MSC-EVs could improve the antisenescence, proliferation, migration, and antiapoptotic abilities of HG-induced senescent HDFs.

### Detection of miRNAs in P-MSC-EVs and the prediction of their targets

**Figure 4:**
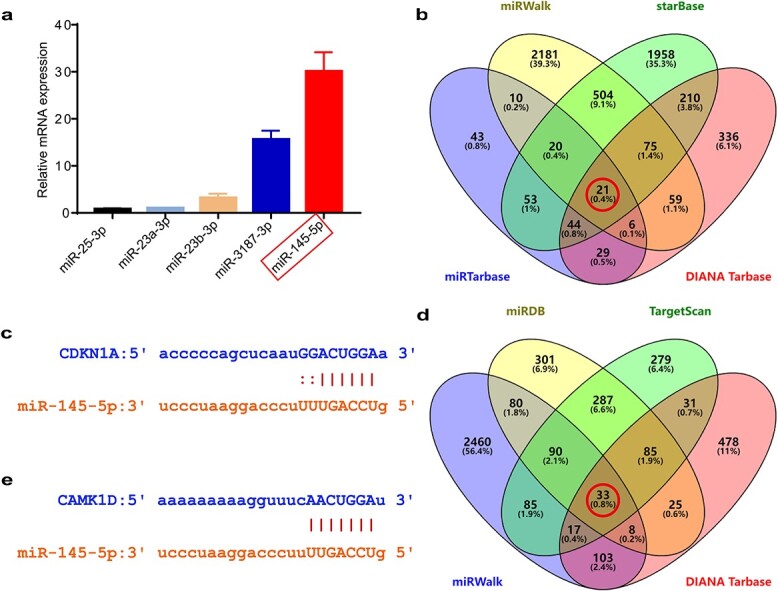
Detection of miRNAs in P-MSC-EVs and prediction of their targets. (**a**) Expression detection of the selected miRNAs with RT-qPCR. n = 3 per group. (**b**) Venn diagram for the intersection of putative miR-145-5p targets predicted using miRwalk, starBase, miRTarbase and DIANA Tarbase. A total of 21 co-predicted genes (red circle) were found including CDKN1A. (**c**) Putative miR-145-5p binding sites in the 3′-UTRs of CDKN1A. (**d**) Venn diagram for the intersection of putative miR-145-5p targets predicted using miRDB, Targetscan, miRwalk and DIANA Tarbase. A total of 33 co-predicted genes (red circle) were identified including CAMK1D. (**e**) Putative miR-145-5p binding sites in the 3′-UTRs of CAMK1D. *CAMK1D* Calcium/calmodulin dependent protein kinase 1D, *CDKN1A* Cyclin dependent kinase inhibitor 1A, *P-MSC-EVs* extracellular vesicles derived from human placental mesenchymal stem cells

MiRNAs, which are important cargo in EVs, are able to exert a regulatory effects on a wide array of biological processes by binding with target mRNAs to regulate their expression. To find the candidate miRNAs responsible for improving the functionality of HG-induced HDFs by P-MSC-EVs, a group of well-studied miRNAs, including miR-602, miR-1290, miR-23b-3p, miR-25-3p, miR-23a-3p, miR-3187-3p and miR-145-5p, in P-MSC-EVs were detected by RT-qPCR and were reported to have positive roles in regulating cell function in other tissues [43–57]. The results indicated that miR-145-5p was the most abundant miRNA in P-MSC-EVs (Figure 4a). Previous research has indicated that miR-145-5p enhances the proliferation and migration of fibroblasts and lowered HG-induced apoptosis and oxidative stress in endothelial cells and podocytes [24,25,53,55]. Therefore, we focused on miR-145-5p in EVs for further experiments. By searching miRWalk, starBase, miRTarBase and DIANA Tarbase, 21 genes, including CDKN1A were predicted (Figure 4b and Table S4, see online supplementary material). Figure 4c shows a putative miR-145-5p binding site in the 3′-UTRs of CDKN1A mRNA. By searching miRDB, TargetScan, miRWalk and DIANA Tarbase, 33 genes, including CAMK1D were predicted (Figure 4d and Table S4). Figure 4e shows a putative miR-145-5p binding site in the 3′-UTRs of CAMK1D mRNA. In addition, CDKN1A and CAMK1D have already been experimentally verified in miRTarbase and DIANA Tarbase databases [39,40] and have also been reported as targets of miR-145-5p [54,58].

### P-MSC-EVs improved the function of HG-induced HDFs by transferring miR-145-5p

**Figure 5:**
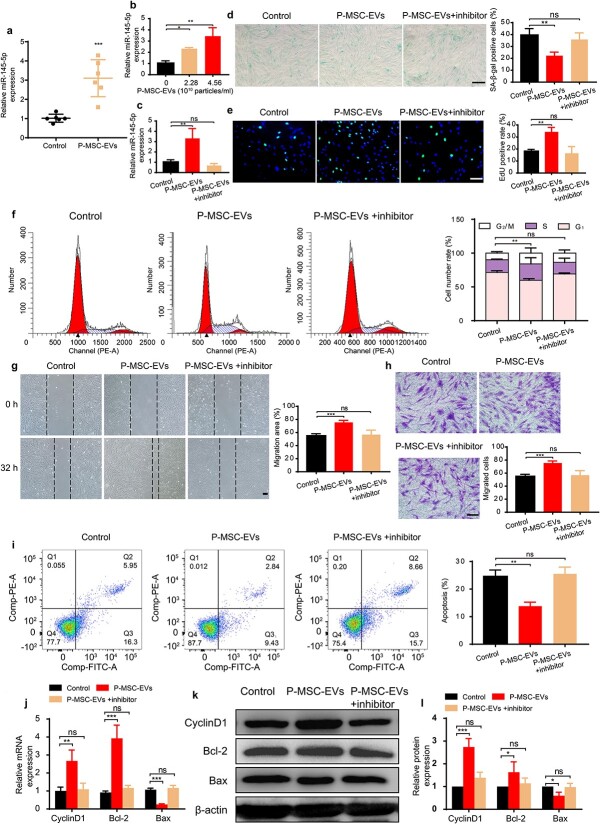
P-MSC-EVs improved the functions of HG-induced senescent HDFs via transferring miR-145-5p. (**a**) Effect of P-MSC-EVs derived from different healthy volunteers on the miR-145-5p expression in HG-induced senescent HDFs. n = 6 per group. (**b**) Effect of the two concentrations of P-MSC-EVs on miR-145-5p expression level in HG-induced senescent HDFs. n = 3 per group. (**c**) MiR-145-5p inhibitors counteracted the overexpression of miR-145-5p in HG-induced senescent HDFs treated with P-MSC-EVs. n = 3 per group. (**d**) SA-β-gal expression in HDFs treated with P-MSC-EVs was decreased by miR-145-5p inhibitors. Scale bar: 200 μm. n = 3 per group. (**e**) Proliferation of HG-induced HDFs treated with P-MSC-EVs was inhibited by miR-145-5p inhibitors (green: EdU staining; blue: Hoechst staining). Scale bar: 100 μm. n = 4 per group. (**f**, **i**) Effect of miR-145-5p inhibitors on cell cycle distribution and apoptosis of HDFs treated with P-MSC-EVs. n = 3 per group. (**g**, **h**) Effect of miR-145-5p inhibitors on the migration of HDFs treated with P-MSC-EVs was evaluated by scratch assay (scale bar: 200 μm) and transwell assay (scale bar: 100 μm). n = 4 per group. (**j**–**l**) MiR-145-5p inhibitors decreased the expression of cyclin D1 and Bcl-2 and increased Bax expression in HDFs treated with P-MSC-EVs. n = 3 per group. Compared with the control group, ^*^p < 0.05; ^*^^*^p < 0.01; ^*^^*^^*^p < 0.001; *ns* no significance. *P-MSC-EVs* extracellular vesicles derived from human placental mesenchymal stem cells, *HDFs *human dermal fibroblasts, *HG* high glucose

To verify the role of miR-145-5p in the P-MSC-EV-mediated functional improvements of HG-induced HDFs, we initially confirmed the increased expression of miR-145-5p in HG-induced HDFs treated with P-MSC-EVs (Figure 5a, b). MiR-145-5p inhibitors were used to knock down miR-145-5p expression (Figure 5c) in HG-induced HDFs after treatment with P-MSC-EVs. Subsequently, a series of functional assays were conducted to test the regenerative effects of P-MSC-EV-derived miR-145-5p. Figure 5d shows that miR-145-5p inhibitors could block the effect of P-MSC-EVs on SA-β-gal expression in HDFs. EdU incorporation and cell cycle assays were carried out to evaluate the proliferation of HDFs, and the results showed that EdU-positive HDFs and cells in S and G_2_/M phase were increased after P-MSC-EV stimulation but were reduced by the transfection of miR-145-5p inhibitors (Figure 5e, f). These results were also confirmed by the CCK8 cell viability assay (Figure S3, see online supplementary material). Next the migratory ability of HG-induced HDFs was attenuated after miR-145-5p inhibition, as shown in in Figure 5g, h. We further quantified the apoptosis rate and found that the effect of P-MSC-EV-induced protection against apoptosis was diminished (Figure 5i). In addition, transfection of the miR-145-5p inhibitors resulted in decreased cyclin D1 and Bcl-2 expression and increased Bax expression, verifying that the enhanced proliferation and antiapoptotic effects on HDFs induced by P-MSC-EVs were partially reversed by the miR-145-5p inhibitors (Figure 5j–l). Collectively, these data demonstrate that miR-145-5p in P-MSC-EVs exert a protective effects on HG-induced senescent HDFs.

### MiR-145-5p mimicked the effects of P-MSC-EVs to improve the function of HG-induced HDFs

**Figure 6:**
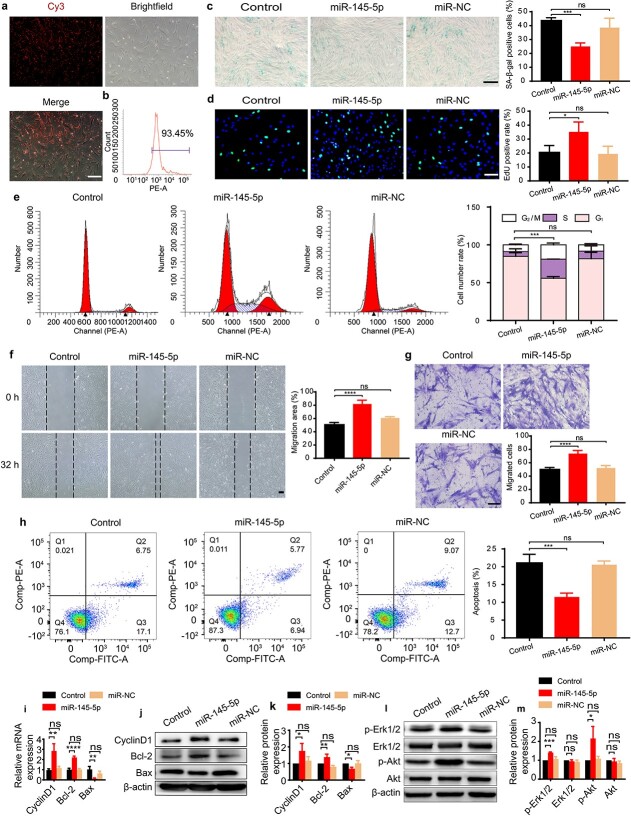
MiR-145-5p mimicked the effects of P-MSC-EVs to improve the function of HG-induced HDFs. (**a**) The transfection efficiency of miR-145-5p mimics in HG-induced senescent HDFs after 12 h. (**b**) The efficiency of Cy3 expression in transfected cells was examined by flow cytometry. (**c**) Effect of miR-145-5p mimics on SA-β-gal expression in HDFs. Scale bar: 200 μm. n = 3 per group. (**d**) Cell proliferation was measured by EdU incorporation assay (green: EdU staining; blue: Hoechst staining). Scale bar: 100 μm. n = 4 per group. (**e**, **h**) Cell cycle and cell apoptosis were quantified by flow cytometry. n = 3 per group. (**f**, **g**) Migratory abilities were evaluated by scratch assay (scale bar: 200 μm) and transwell assay (scale bar: 100 μm). n = 4 per group. (**i**–**k**) Cyclin D1, Bcl-2 and Bax expression were quantified by RT-qPCR and western blotting. n = 3 per group. (**l**, **m**) Expression levels of p-Erk1/2 and p-Akt in HG-induced senescent HDFs and the quantification results (m). n = 3 per group. Compared with the control group, ^*^p < 0.05; ^*^^*^p < 0.01; ^*^^*^^*^p < 0.001; ^*^^*^^*^^*^p < 0.0001; *ns* no significance. *P-MSC-EVs* extracellular vesicles derived from human placental mesenchymal stem cells, *HDFs* human dermal fibroblasts, *HG* high glucose

Subsequently, we further investigated whether miR-145-5p could mimic the effects of P-MSC-EVs on HG-induced HDFs. MiR-145-5p mimics or miR-NC were transfected into HG-induced HDFs, and the transfection rate was shown to be 93.45% (Figure 6a, b). After transfection, SA-β-gal staining results verified the beneficial effects of miR-145-5p on improving HDF senescence (Figure 6c). The EdU and CCK-8 results confirmed the ability of miR-145-5p to enhance the proliferation of HG-induced senescent HDFs (Figure 6d and Figure S4, see online supplementary material). Consistently, in miR-145-5p mimic-treated HDFs, more cells were in the S and G_2_/M phases than in the control and miR-NC groups (Figure 6e). The results of the cell migration assay showed that HG-induced HDFs transfected with miR-145-5p mimics migrated faster than cells in control and miR-NC groups (Figure 6f, g). As expected, fewer apoptotic cells were found in the miR-145-5p mimics group (Figure 6h). Additionally, we observed that miR-145-5p mimics upregulated cyclin D1 and Bcl-2 expression and downregulated Bax expression at the mRNA and protein levels (Figure 6i–k). Numerous studies have reported that MSC-EV-derived miRNAs could enhance wound healing at least in part by activating the Erk/Akt signaling pathway [59,60]. Therefore, the expression levels of Erk1/2, p-Erk1/2, Akt and p-Akt were detected to determine whether miR-145-5p could activate the Erk/Akt signaling pathway. As expected, miR-145-5p mimics notably increased the phosphorylation of Erk1/2 and Akt, although there was no significant change in the expression of Erk1/2 or Akt (Figure 6l, m). In summary, miR-145-5p mimics could activate the Erk/Akt signaling pathway and mimic the effects of P-MSC-EVs on improving the functions of HG-induced senescent HDFs in terms of inhibiting senescence and apoptosis and inducing proliferation and migration *in vitro*.

### MiR-145-5p improved the function of HG-induced HDFs by targeting CDKN1A to activate the Erk/Akt pathway

**Figure 7:**
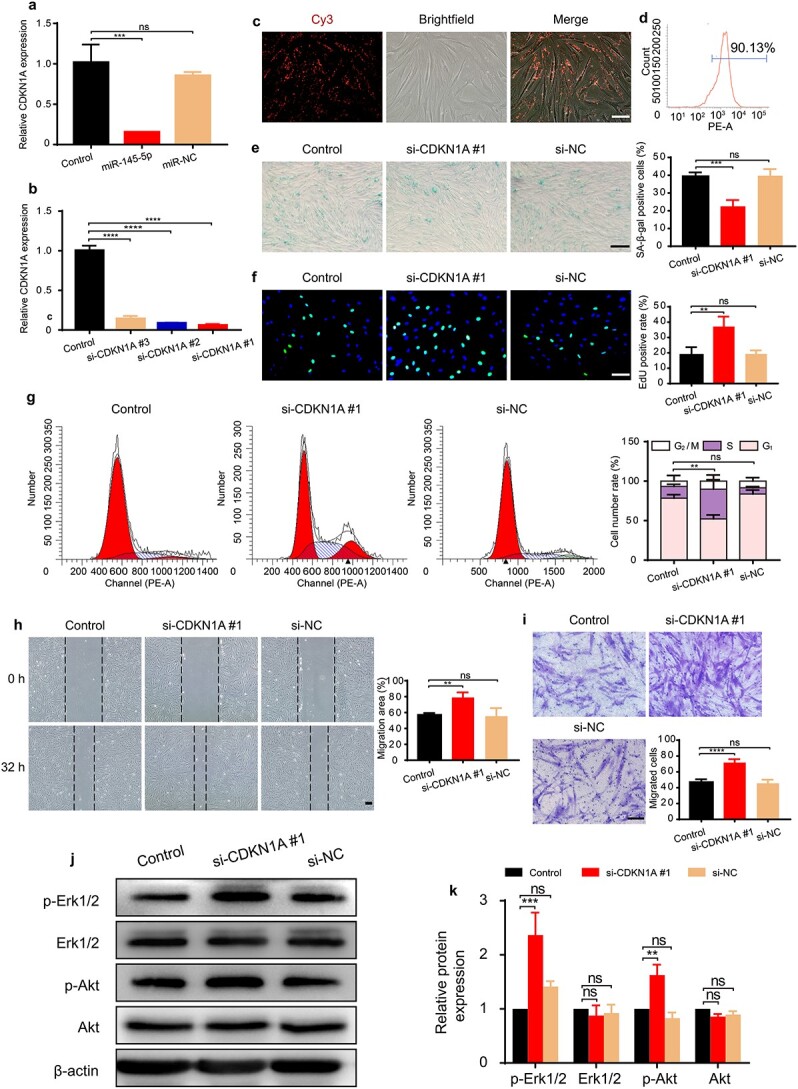
MiR-145-5p enhanced the proliferation and migration of HG-induced HDFs via targeting CDKN1A/Erk/Akt signaling. (**a**) The effect of miR-145-5p on the expression of CDKN1A was assessed by RT-qPCR. n = 3 per group. (**b**) The inhibitory efficiency of the siRNAs targeting CDKN1A was verified by RT-qPCR. Si-CDKN1A #1 was used in subsequent experiments. n = 3 per group. (**c**, **d**) The transfection efficiency of si-CDKN1A #1 in HG-induced senescent HDFs after 12 h was detected by flow cytometry. (**e**) SA-β-gal expression in HG-induced senescent HDFs with different treatments. Scale bar: 200 μm. n = 3 per group. (**f**) The effect of si-CDKN1A #1 on the proliferation of HG-induced senescent HDFs was measured by EdU assay (green: EdU staining; blue: Hoechst staining). Scale bar: 100 μm. n = 4 per group. (**g**) Cell cycle distribution detected by flow cytometry. n = 3 per group. (**h**, **i**) Migratory abilities were evaluated by scratch assay (scale bar: 200 μm) and transwell assay (scale bar: 100 μm). n = 4 per group. (**j**, **k**) Effects of si-CDKN1A #1 on the protein expression levels of p-Akt and p-Erk1/2 in HG-induced senescent HDFs were evaluated by western blotting. n = 3 per group. Compared with the control group, ^*^^*^p < 0.01; ^*^^*^^*^p < 0.001; ^*^^*^^*^^*^p < 0.0001; *ns* no significance. *CDKN1A* cyclin dependent kinase inhibitor 1A, *HDFs* human dermal fibroblasts, *HG* high glucose, *p-Akt *Phosphorylated Akt, *p-Erk* Phosphorylated Erk

CDKN1A was further confirmed to be the target gene of miR-145-5p (Figure 7a). To test whether knocking down the expression of CDKN1A could achieve comparable effects as miR-145-5p on cell function, si-CDKN1A #1 was selected to inhibit the expression of CDKN1A in HG-induced HDFs (Figure 7b). The transfection rate was shown to be 90.13% (Figure 7c, d). Figure 7e shows reduced SA-β-gal expression in HDFs transfected with si-CDKN1A #1. The EdU and CCK-8 results indicated that si-CDKN1A #1-transfected HDFs exhibited higher cell proliferation (Figure 7f and Figure S5, see online supplementary material). Correspondingly, we found the majority of si-CDKN1A #1-transfected HDFs in the S and G_2_/M phases (Figure 7g). Furthermore, the migration ability of HDFs detected by scratch and transwell assays was also increased in the si-CDKN1A #1 group (Figure 7h, i). Unexpectedly, we did not find a decrease in apoptosis after ransfection with si-CDKN1A (Figure S6, see online supplementary material), indicating that other target genes of miR-145-5p may be involved in regulating the antiapoptotic effects on HG-induced HDFs. CDKN1A is involved in arresting cell cycle progression and has been reported to be a negative regulator of the Erk/Akt signaling pathway in some tumor cells [61]. Consistent with their reports, CDKN1A inhibition induced significant increases in the phosphorylation of Erk1/2 and Akt in our study, indicating that activation of the Erk/Akt pathway may be the underlying mechanism by which CDKN1A inhibition enhances fibroblast function (Figure 7j, k).

**Figure 8:**
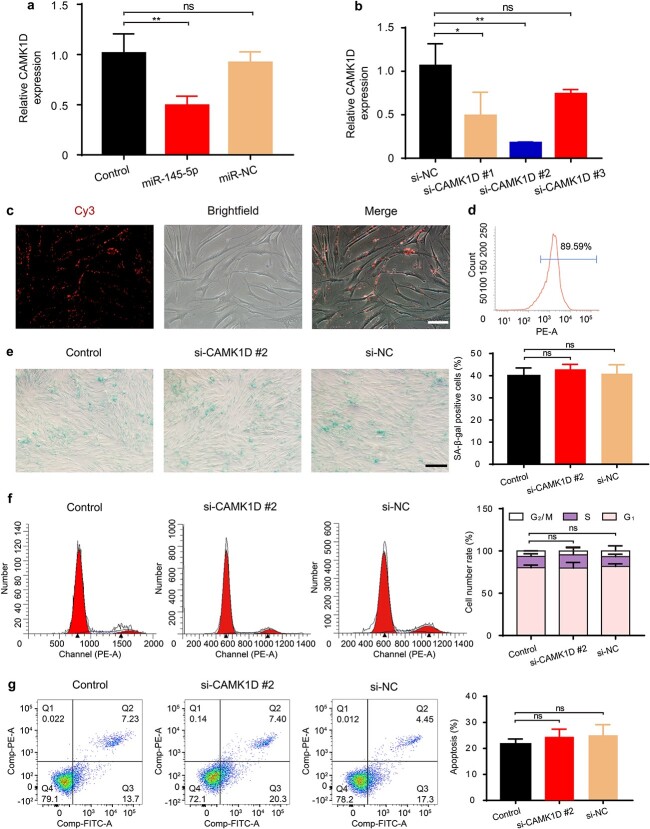
CAMK1D inhibition had no effects on the function of HG-induced senescent HDFs. (**a**) The effect of miR-145-5p on the expression of CDKN1A was assessed by RT-qPCR. n = 3 per group. (**b**) The inhibitory efficiency of the siRNAs targeting CAMK1D was verified by RT-qPCR. Si-CAMK1D #2 was used in subsequent experiments. n = 3 per group. (**c**) Si-CAMK1D #2 transfected into HG-induced senescent HDFs after 12 h was observed. (**d**) Transfection efficiency was examined by flow cytometry. (**e**) SA-β-gal expression in HDFs treated with si-CAMK1D #2 or si-NC. Scale bar: 200 μm. n = 3 per group. (**f**, **g**) Cell cycle distribution and cell apoptosis rate detected by flow cytometry. n = 3 per group. Compared with the control group, *ns* no significance. *CDKN1A* cyclin dependent kinase inhibitor 1A, *CAMK1D* calcium/calmodulin dependent protein kinase 1D, *HDFs* human dermal fibroblasts, *HG* high glucose

CAMK1D was confirmed as another target gene of miR-145-5p (Figure 8a). Similarly, we chose the most effective siRNA, si-CAMK1D #2, to inhibit CAMK1D expression in functional assays of HG-induced HDFs (Figure 8b). The transfection rate was shown to be 89.59% (Figure 8c, d). Disappointingly, we found that the transfection of si-CAMK1D #2 had no effects on cell senescence, cell cycle or cell apoptosis of HG-induced HDFs (Figure 8e–g), indicating that CAMK1D was not involved in the functional improvements of HG-induced HDFs induced by miR-145-5p derived from P-MSC-EVs.

These results indicate that miR-145-5p is responsible for the P-MSC-EV-induced functional improvements of HG-induced HDFs by targeting CDKN1A to activate the Erk/Akt pathway.

### MiR-145-5p mimicked the effects of P-MSC-EVs to promote wound healing *in vivo*

**Figure 9:**
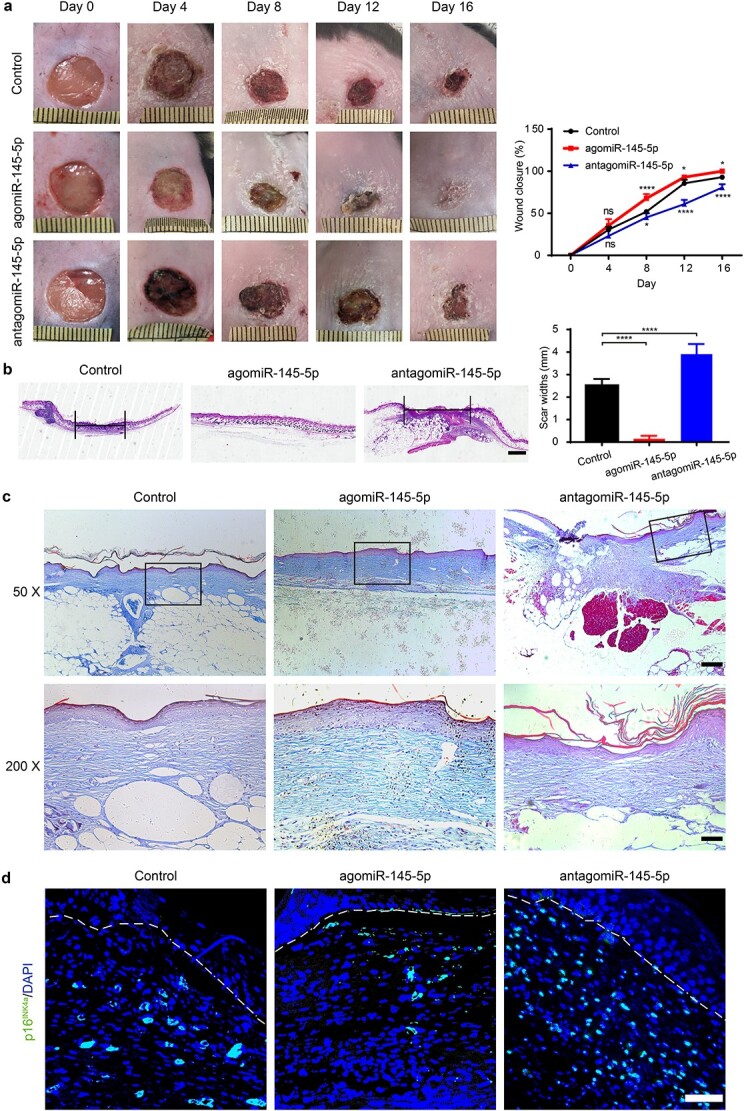
MiR-145-5p mimicked the effects of P-MSC-EVs to promote wound healing *in vivo*. (**a**) General view and the rate of wound closure with different treatments at days 0, 4, 8, 12 and 16 after wounding, n = 5 per group. (**b**) H&E staining of wound sections treated with PBS (control), agomiR-145-5p and antagomiR-145-5p at day 16 after the operation. Scale bar: 1 mm. (**c**) Masson’s trichrome staining at day 16 post-wounding. Collagen fiber was stained in blue. Scale bar: 2 mm, 500 μm. (**d**) Representative images of fibroblastic p16INK4a immunofluorescent staining in skin wounds from diabetic mice treated with PBS, agomiR-145-5p and antagomiR-145-5p on Day 16 after the operation. Scale bar: 50 μm. Compared with the control group, ^*^p < 0.05; ^*^^*^^*^^*^p < 0.0001; *ns* no significance, *P-MSC-EVs* extracellular vesicles derived from human placental mesenchymal stem cells,* PBS *phosphate-buffered saline, *H&E* hematoxylin and eosin

A previous experiment revealed that miR-145-5p could mimic the effects of P-MSC-EVs to improve the function of HG-induced HDFs. Next, we assessed the effects of miR-145-5p on diabetic wound healing. Full-thickness cutaneous wounds were made on the backs of diabetic mice and were injected with PBS, agomiR-145-5p, and antagomiR-145-5p every two days after wounding. *In vivo* image analysis confirmed the continuous retention of agomiR-145-5p two days after injection (Figure S7, see online supplementary material). Additionally, as shown in Figure 9a, the area of the wounds in the agomiR-145-5p group was significantly decreased on Days 8, 12, and 16 after the operation relative to the other groups. Furthermore, the narrowest scar widths, as well as much longer, thicker, and better-organized fibers, were observed in wounds treated with agomiR-145-5p than in the control and antagomiR-145-5p groups on Day 16 after wounding (Figure 9b, c). Moreover, we evaluated p16INK4a expression at the wound site, and the results confirmed that agomiR-145-5p was able to improve the fibroblast senescence state *in vivo* (Figure 9d). Collectively, these data demonstrate that miR-145-5p could mimic the effects of P-MSC-EVs to promote diabetic wound healing and collagen deposition and improve fibroblast senescence, highlighting its great potential in diabetic wound treatment.

## Discussion

In this study, we first proved that local injection of P-MSC-EVs into cutaneous wound sites in diabetic mice leads to rapid wound closure and better-organized collagen deposition. *In vitro*, P-MSC-EVs could be internalized by HG-induced senescent HDFs and subsequently enhanced the cell antisenescence, proliferation, migration, and antiapoptotic abilities of HDFs. Next, the potential underlying mechanisms were explored, and miR-145-5p was shown to be abundant in P-MSC-EVs, which could be delivered into HDFs to activate the Erk/Akt signaling pathway by targeting CDKN1A. With agomirs and antagomirs, we further confirmed that miR-145-5p plays a key role in the positive effects of P-MSC-EVs on diabetic wounds.

Diabetic wounds are associated with great healing difficulties, high costs, severe disability due to amputation and intense care. Over the past decades, accumulating evidence has indicated that cellular senescence is one of the causes of diabetic wound healing [62]. Cellular senescence is traditionally defined as permanent cell growth arrest and is divided into two main types: telomere-dependent replicative senescence and stress-induced premature senescence [63]. Many studies have proven that persistent hyperglycaemia is involved in accelerating the shortening of telomere length and inducing stress-induced premature senescence, eventually giving rise to large-scale cellular senescence. These senescent cells were characterized by impaired cell proliferation, migration, and antiapoptotic abilities, thereby inhibiting wound healing [32,34]. HDFs serve as one of the main repair cells, and functional defects in any biological behavior can lead to healing failure. In the present study, we confirmed that HG at a concentration of 35 mM could induce HDF senescence. Moreover, HG-induced HDFs exhibited lower proliferation, migration, and antiapoptotic abilities, which was consistent with previous reports [32,42]. Therefore, it is of pivotal importance to protect HDFs from HG insult to achieve rapid wound healing.

Stem cells derived from different tissues have been reported to have positive effects on diabetic wound repair. However, the actual effects of stem cell transplantation were confined to donor condition and functional debilitation after long-term culture and successive passages [64]. The application of these cells poses uncertain risks of immune rejection, tumorigenicity, and ethical issues [65]. Recently, many studies have proved that the beneficial effects of stem cells are mainly attributable to their exocrine function rather than direct differentiation into target cells [10,59]. EVs are key paracrine factors secreted from stem cells that can regulate the functions of target cells during wound repair [66–68]. MSC-EVs originating from bone marrow, umbilical cord, placenta, and adipose tissue, as well as EVs derived from human amniotic epithelial cells [12,59,69,70], have been reported to accelerate wound healing and have regenerative and protective effects on wound repair cells, especially on fibroblasts [71,72]. In the current study, we demonstrated the beneficial effects of P-MSC-EVs on diabetic wound healing and functional improvements of HG-induced senescent HDFs. Likewise, P-MSC-EVs were reported to stimulate angiogenesis in ischaemic diseases [12] and exert therapeutic effects on Duchenne muscular dystrophy patients [73].

We investigated the mechanism responsible for the positive effects of P-MSC-EVs on HG-induced senescent HDFs. EVs contain a variety of components in addition to miRNAs that can protect miRNAs from extracellular degradation. MiRNAs are involved in regulating multiple biological processes in type II diabetes including insulin secretion, immune inflammatory response, angiogenesis and diabetic wound healing [14,74]. In this study, candidate miRNAs within P-MSC-EVs were identified using RT-qPCR. We first reported that miRNA-145-5p was highly enriched in P-MSC-EVs. Currently, during tumor treatment, miR-145-5p has been reported to have controversial effects. For example, miR-145-5p serves as a tumor suppressor in melanoma, tongue and laryngeal squamous cell carcinoma, hepatocellular carcinoma, colorectal cancer, pancreatic ductal adenocarcinoma, and lung cancer by inhibiting the proliferation, migration, and antiapoptotic status of tumor cells [17,75–80], while Zhou *et al*. found that miR-145-5p could enhance the proliferation, migration, and invasion of Wilms’ tumor cells [81]. Apart from mediating tumorigenic functions, miR-145-5p was found to be down-regulated in hypertrophic scar tissues, psoriatic lesional skin, hepatopulmonary syndrome tissues, lung tissues of smokers and atherosclerotic plaques of atherosclerosis mice, and miR-145-5p overexpression was able to attenuate the proliferation, migration, and antiapoptotic abilities of hypertrophic scar fibroblasts, keratinocytes, pulmonary microvascular endothelial cells, astrocytes, and cardiac cells [82–90]. Nevertheless, miR-145-5p overexpression has also been demonstrated to be capable of enhancing the proliferation and migration of trophoblasts cells, and airway smooth muscle cells *in vitro* [91–93]. Moreover, Yang *et al*. and Condorelli *et al*. found that miR-145-5p overexpression improved the viability and migration of lung fibroblasts and recessive dystrophic epidermolysis bullosa skin fibroblasts and led to the establishment and maintenance of fibrotic traits of contraction, which was accompanied by upregulated expression of α-smooth muscle actin [53,55]. Additionally, miR-145-5p could be induced by TGF-β and dihydroartemisinin. However, dihydroartemisinin could significantly reverse the proliferation and fibrosis of tenon fibroblasts caused by TGF-β [94,95]. These results indicate that miR-145-5p may be a potential target in the development of novel gene therapies to treat pathological fibrotic diseases.

In addition, miR-145-5p is involved in exerting anti-inflammation or protective effects on kidney disease [96,97], spinal cord injury [98,99], neuropathic pain in chronic constriction injury [100], abdominal aortic aneurysm [101], myocardial ischaemia and ischaemia–reperfusion injury [89,102–104], acute lung injury [105,106] and chronic obstructive pulmonary disease [88,107–109], which are accompanied by negatively regulating proinflammatory cytokine release [including interleukin-1α (IL-1α), IL-2, IL-6 and tumor necrosis factor-α (TNF-α)], downregulatng Bax and cleaved caspase-3, upregulating Bcl-2 and reducing reactive oxygen species. Moreover, miR-145-5p could also alleviate the inflammation response and apoptosis in endothelial cells induced by cigarette smoke extract, particulate matter 2.5 (PM_2.5_) and hypoxia/reoxygenation [88,110–112]. Conversely, it has also been reported that miR-145-5p could exacerbate the immune response, but this is not clear [113,114].

Recently, several studies showed that miR-145-5p expression was highly associated with diabetes occurrence [19], diabetic retinopathy progression [20], diabetic kidney diseases [21], diabetic foot ulcers [22], HG-induced injury [23–25], and a high-fat, high-saturated diet [29]. In addition, miR-145-5p was found to be upregulated in exosomes derived from HG-induced retinal ganglion cells, and mesangial cells [20,30] but was downregulated in exosomes derived from HG-treated vascular smooth muscle cells [23]. MiR-145-5p inhibition protected these cells from HG-induced suppression of cell viability and increased apoptosis with decreases in proinflammatory cytokines, including TNF-α and IL-6 [20,23]. In contrast, miR-145-5p expression was suppressed by HG in human renal tubular epithelial cells, and miR-145-5p overexpression decreased the apoptosis rates of renal tubular epithelial cells induced by HG, which was accompanied by decreased expression of Bax, IL-1β and IL-6 and elevated expression of Bcl-2 [115].

Thus, the controversial effects of miR-145-5p may be cellular or tissue context-dependent. However, the effects of miR-145-5p derived from P-MSC-EVs on HG-induced HDFs and diabetic wounds remain unclear. We incubated HG-induced HDFs with P-MSC-EVs and found that the expression of MiR-145-5p was markedly enhanced. miR-145-5p inhibitors could reverse the positive effects of P-MSC-EVs on HG-induced HDFs. MiR-145-5p overexpression could mimic the effects of P-MSC-EVs to improve the function of HG-induced HDFs. Moreover, agomiR-145-5p could simulate the effects of P-MSC-EVs to promote diabetic wound healing and collagen deposition, as well as alleviate cell senescence. Based on these findings, we suggest that miR-145-5p is one of the major mediators of P-MSC-EV-induced functional improvements of HG-induced senescent HDFs, thereby accelerating diabetic wound healing.

Subsequently, the target genes and the downstream signaling pathways of miR-145-5p were explored. CDKN1A is a cyclin-dependent kinase inhibitor. During cell proliferation, CDKN1A functions as a cell cycle regulator at the G_1_ phase [116]. After transfection with miR-145-5p mimics, the expression of CDKN1A was markedly decreased in HG-induced HDFs. Furthermore, knocking down the expression of CDKN1A could simulate the effects of P-MSC-EVs and miR-145-5p mimics to enhance the antisenescence, proliferation, and migration abilities, but not the antiapoptotic ability, suggesting other target genes of miR-145-5p in regulating cell apoptosis. In addition, our study also confirmed the connection between CDKN1A and the activated signaling pathways in recipient cells. Previous studies reported that TGF-β/Smad2/3/4 [106,107,112], p53 pathway [103], Akt, nuclear factor kappa B and the c-Jun N-terminal kinase pathway [96,98–100,105,111] were frequently activated and involved in miR-145-5p-miedated protection. Among them, the Erk/Akt signaling pathway has been reported to be involved in EV-induced wound healing by optimizing cellular functions [59,60]. CDKN1A is a negative regulator of the Erk/Akt pathway in tumour cells [61]. Consistent with these reports, our results showed that downregulation of CDKN1A increased the expression of p-Erk1/2 and p-Akt. We conclude that miR-145-5p derived from P-MSC-EVs optimized the functions of HG-induced HDFs by targeting CDKN1A to activate the Erk/Akt pathway. The *in vivo* experiment showed that topical treatment with agomiR-145-5p promoted wound healing and collagen deposition and decreased p16INK4a expression *in vivo*, whereas these effects were markedly attenuated by the injection of antagomiR-145-5p in wound sites. Because pro-healing miR-145-5p was already presented in skin tissues, fibroblasts, and EVs [19,28,117] and was eliminated by antagomiR-145-5p, effective miR-145-5p inhibitors *in vivo*. Although we concluded that the Erk/Akt signalling pathway activated by miR-145-5p/CDKN1A and contributed to the beneficial effects of P-MSC-EVs on diabetic wound healing, the present study has some limitations. First, we did not detect miR-145-5p and CDKN1A expression after treatments *in vivo* to confirm the *in vitro* results. Second, we failed to find underlying direct or indirect targets of miR-145-5p responsible for the antiapoptotic effects on HG-induced senescent HDFs. Third, we did not assess the extent of the transcriptional levels of certain key proteins in the Erk/Akt pathway in the wound-healing model. Lastly, experiments with higher concentrations of P-MSC-EVs needed to be performed to observe whether this could induce better pro-regenerative effects in diabetic wound healing.

## Conclusions

In summary, our findings demonstrate that P-MSC-EVs can effectively promote diabetic wound healing by inhibiting senescence and apoptosis and inducing proliferation and migration of HG-induced senescent HDFs. These therapeutic effects of P-MSC-EVs are miR-145-5p dependent. Moreover, miR-145-5p could directly target CDKN1A to activate the Erk/Akt signaling pathway. It will also be of great importance to determine whether P-MSC-EVs has positive effects on other cell types involved in wound healing. Our findings confirmed that the use of P-MSC-EVs encapsulating therapeutic miRNAs may be a promising therapeutic approach for diabetic wound treatment in the future.

## Abbreviations

agomiR-145-5p: miR-145-5p agomir; antagomiR-145-5p: miR-145-5p antagomir; CAMK1D: Calcium/calmodulin dependent protein kinase 1D; CCK-8: Cell counting kit-8; CDKN1A: Cyclin dependent kinase inhibitor 1A; Cy3: Cyanine 3; Erk: Extracellular signal regulated kinase; EVs: Extracellular vesicles; FBS: Fetal bovine serum; HDFs: Human dermal fibroblasts; HG: high glucose; H&E: hematoxylin and eosin; IL-1α: Interleukin-1α; miRNAs: microRNAs; miR-NC: Mimic negative control; NTA: Nanoparticle tracking analysis; MSC: Mesenchymal stem cell; p-Akt: Phosphorylated Akt; PBS: phosphate-buffered saline; p-Erk: Phosphorylated Erk; PI: Propidium iodide; P-MSC-EVs: EVs derived from human placental mesenchymal stem cells; RT-qPCR: real-time quantitative PCR; siRNA: Small interfering RNA; TNF-α: Tumor necrosis factor-α; TGF-β: Transforming growth factor-β; TSG101: Tumor susceptibility gene 101; UTR: Untranslated region.

## Funding

This work was supported by the National Nature Science Foundation of China (82172211, 81830064, 82172231, 81901971), National Key Research and Development Programs of China (2022YFA1104303), the CAMS Innovation Fund for Medical Sciences (CIFMS, 2019-I2M-5-059), the Military Medical Research and Development Projects (AWS17J005, 2019–126) and the Military Medical Science and Technology Youth Training Program (21QNPY128).

## Data availability

Data supporting the results of this study can be obtained from the corresponding author upon reasonable request.

## Authors' contributions

JLS, QW, CPZ, XBF and HHL were involved in the study design. JLS and QW mainly conducted the experiments and the statistical analyses. HM, YXW, YHZ and WHZ participated in the surgery of mice. WZH and QKL participated in data analysis and interpretation. JLS wrote the first draft of the manuscript. QW and KM revised the manuscript. CPZ, XBF and HHL participated in supervision of this research. All authors read and approved the final manuscript.

## Ethics approval and consent to participate

The study involving human participants and tissue was performed in line with the Helsinki Declaration and approved by the Ethics Committee of Fourth Medical Center of the PLA General Hospital (ethical permission number: 2022KY063-KS001). All animal procedures were approved under the guidelines of the Ethics Committee of the Laboratory Animal Research Center of Beijing Sibeifu Laboratory Animal Technology (approval no. AWE2021091501).

## Conflicts of interest

The authors declare that they have no competing interests.

## Supplementary Material

Supplementary_file_1_tkad010

Revised_Supplementary_file_2_20230312_tkad010
