# Supplementary material for: P-MSC-derived extracellular vesicles facilitate diabetic wound healing via miR-145-5p/ CDKN1A-mediated functional improvements of high glucose-induced senescent fibroblasts
Source: Burns Trauma. 2023 Oct 18;11:tkad010. doi: 10.1093/burnst/tkad010 (PMC10583213; doi:10.1093/burnst/tkad010)
Supplement: Supplementary_file_1_tkad010 [file supplementary_file_1_tkad010.docx]

**Supplementary tables**

| **Table S1. The sequences of miRNA mimic, miRNA inhibitor, siRNA, agomiRNA and antagomiRNA** | |
| --- | --- |
| **Gene** | **Sequences** |
| hsa-miR-145-5p-mimic | sense: GUCCAGUUUUCCCAGGAAUCCCU  antisense: GGAUUCCUGGGAAAACUGGACUU |
| hsa-mimic-NC | sense: UUCUCCGAACGUGUCACGUTT  antisense: ACGUGCACGUUCGGAGAATT |
| hsa-miR-145-5p-inhibitor | 5′-AGGGAUUCCUGGGAAAACUGGAC-3′ |
| hsa-inhibitor-NC | 5′-CAGUACUUUUGUCUAGUACAA-3′ |
| hsa-CDKN1A 1#-siRNA | sense: GAUGGAACUUCGACUUUGUTT  antisense: ACAAAGUCGAAGUUCCAUCTT |
| hsa-CDKN1A 2#-siRNA | sense: CCUCUGGCAUUAGAAUUAUTT  antisense: AUAAUUCUAAUGCCAGAGGTT |
| hsa-CDKN1A 3#-siRNA | sense: CAGGCGGUUAUGAAAUUCATT  antisense: ACGUGACACGUUCGGAGAATT |
| hsa-CAMK1D 1#-siRNA | sense: GGCAACUGGCAAGCUCUUUTT  antisense: AAAGAGCUUGCCAGUUGCCTT |
| hsa-CAMK1D 2#-siRNA | sense: CCAGAAACCUUACAGCAAATT  antisense: UUUGCUGUAAGGUUUCUGGTT |
| hsa-CAMK1D 3#-siRNA | sense: GCAAAUGGAGACAAGCAUUTT  antisense: AAUGCUUGUCUCCAUUUGCTT |
| NC-siRNA | sense: UUCUCCGAACGUGUCACGUTT  antisense: ACGUGACACGUUCGGAGAATT |
| hsa-agomiR-145-5P | sense: GUCCAGUUUUCCCAGGAAUCCCU  antisense: GGAUUCCUGGGAAAACUGGACUU |
| antagomiR-145-5p | 5′- GUCCAGUUUUCCCAGGAAUCCCU -3′ |

**Table S2. Primer sequences used for mRNA PCR**

| **Gene (mRNA)** | **Primer sequence (5′ to 3′)** |
| --- | --- |
| hsa-CyclinD1-forward | TTGCCCTCTGTGCCACAGAT |
| hsa-CyclinD1-reverse | TCAGGTTCAGGCCTTGCACT |
| hsa-Bcl-2-forward | GATAACGGAGGCTGGGATGC |
| hsa-Bcl-2-reverse | TCACTTGTGGCCCAGATAGG |
| hsa-Bax-forward | CCCTTTTGCTTCAGGGTTTC |
| hsa-Bax-reverse | GAGACACTCGCTCAGCTTCTTG |
| hsa-CAMK1D-forward | TGCTGTGAAGTGTATCCCTAAGA |
| hsa-CAMK1D-reverse | TCTCAGGACGGCTATCTCATTC |
| hsa-CDKN1A-forward | GTGGGGTTATCTCTGTGTTAG GG |
| hsa-CDKN1A-reverse | CCCTGTCCATAGCCTCTA CTGC |
| hsa-β-actin-forward | GAGCGCGGCTACAGCTT |
| hsa-β-actin-reverse | TCCTTAATGTCACGCACGATTT |

| **Table S3.**  **PCR primer sequences for miRNA** | |
| --- | --- |
| **Gene (miRNA)** | **Primer sequences (5’-3’)** |
| The same antisense primer was used for all miRNA PCR | GTGCAGGGTCCGAGGT |
| hsa-miR-1290-RT | GTCGTATCCAGTGCAGGGTCCGAGGTATTCGCACTGGATACGACTCCCTG |
| hsa-miR-1290-F | GCGCGCGTGGATTTTTGGAT |
| hsa-miR-602-RT | GTCGTATCCAGTGCAGGGTCCGAGGTATTCGCACTGGATACGACGGGCCG |
| hsa-miR-602-F | CGACACGGGCGACAGCTG |
| hsa-miR-23a-3p-RT | GTCGTATCCAGTGCAGGGTCCGAGGTATTCGCACTGGATACGACGGAAAT |
| hsa-miR-23a-3p-F | GCGCATCACATTGCCAGGG |
| hsa-miR-145-5p-RT | GTCGTATCCAGTGCAGGGTCCGAGGTATTCGCACTGGATACGACAGGGAT |
| hsa-miR-145-5p-F | GCGGTCCAGTTTTCCCAGGA |
| hsa-miR-25-3p-RT | GTCGTATCCAGTGCAGGGTCCGAGGTATTCGCACTGGATACGACTCAGAC |
| hsa-miR-25-3p-F | GCGCCATTGCACTTGTCTCG |
| hsa-miR-3187-3p-RT | GTCGTATCCAGTGCAGGGTCCGAGGTATTCGCACTGGATACGACCCGCGC |
| hsa-miR-3187-3p-F | GCGCTTGGCCATGGGGCT |
| hsa-miR-23b-3p-RT | GTCGTATCCAGTGCAGGGTCCGAGGTATTCGCACTGGATACGACTACCAC |
| hsa-miR-23b-3p-F | GCGCATCCCTGGCAATGTGAT |
| cel-miR-39-RT | GTCGTATCCAGTGCAGGGTCCGAGGTATTCGCACTGGATACGACCAAGCT |
| cel-miR-39-F | GGCCTCACCGGGTGTAAATCAG |
| U6-RT | GTCGTATCCAGTGCAGGGTCCGAGGTATTCGCACTGGATACGACAAAATA |
| U6-F | AGAGAAGATTAGCATGGCCCCTG |

**Table S4. Common predicted targets of CDKN1A and CAMK1D in miRNA-related dabases**

| 21 common predicted targets of CDKN1A in "miRTarbase", "miRWalk", "starBase" and "DIANA Tarbase": | 33 common predicted targets of CAMK1D in "miRWalk", "miRDB", "TargetScan" and "DIANA Tarbase" |
| --- | --- |
| CDKN1A | CAMK1D |
| CLINT1 | ADD3 |
| TMOD3 | VAPA |
| IGF1R | UBE2W |
| CPEB4 | TAGLN2 |
| FZD7 | TPM3 |
| SRGAP1 | IGF1R |
| SERPINE1 | MAPK4 |
| MDM2 | GCLM |
| ADAM17 | ADPGK |
| RTKN | RTKN |
| F11R | ABRACL |
| TPM3 | NEDD9 |
| TMEM9B | CAV2 |
| AP1G1 | CLIP1 |
| ABRACL | G3BP1 |
| SP1 | SLC1A2 |
| ARF6 | SRGAP1 |
| ADD3 | VEZF1 |
| VGLL4 | TMOD3 |
| ZFAND3 | RLIM |
|  | RAB14 |
|  | C5orf15 |
|  | TULP4 |
|  | ARIH1 |
|  | PURA |
|  | PCSK5 |
|  | IPO7 |
|  | ZBTB33 |
|  | ADAM17 |
|  | ARF6 |
|  | SERPINE1 |
|  | AP1G1 |
