# Supplementary material for: P-MSC-derived extracellular vesicles facilitate diabetic wound healing via miR-145-5p/ CDKN1A-mediated functional improvements of high glucose-induced senescent fibroblasts
Source: Burns Trauma. 2023 Oct 18;11:tkad010. doi: 10.1093/burnst/tkad010 (PMC10583213; doi:10.1093/burnst/tkad010)
Supplement: Revised_Supplementary_file_2_20230312_tkad010 [file revised_supplementary_file_2_20230312_tkad010.docx]

**Supplementary figures and figure legends**

**
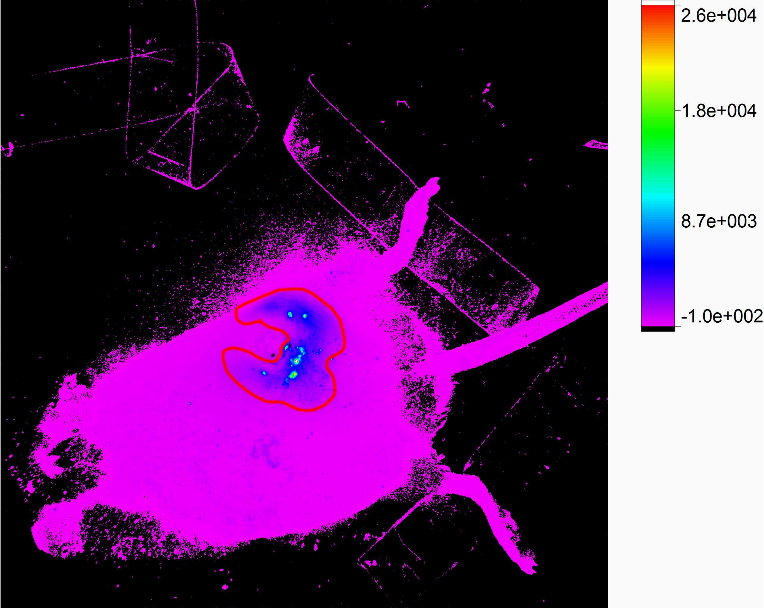
**

**Figure S1. Retention of P-MSC-EVs in skin tissues.** Representative images of P-MSC-EV retention in skin tissues on Day 2 after injection. Blue zone in red circle indicates injected P-MSC-EVs labeled with PKH26.

**
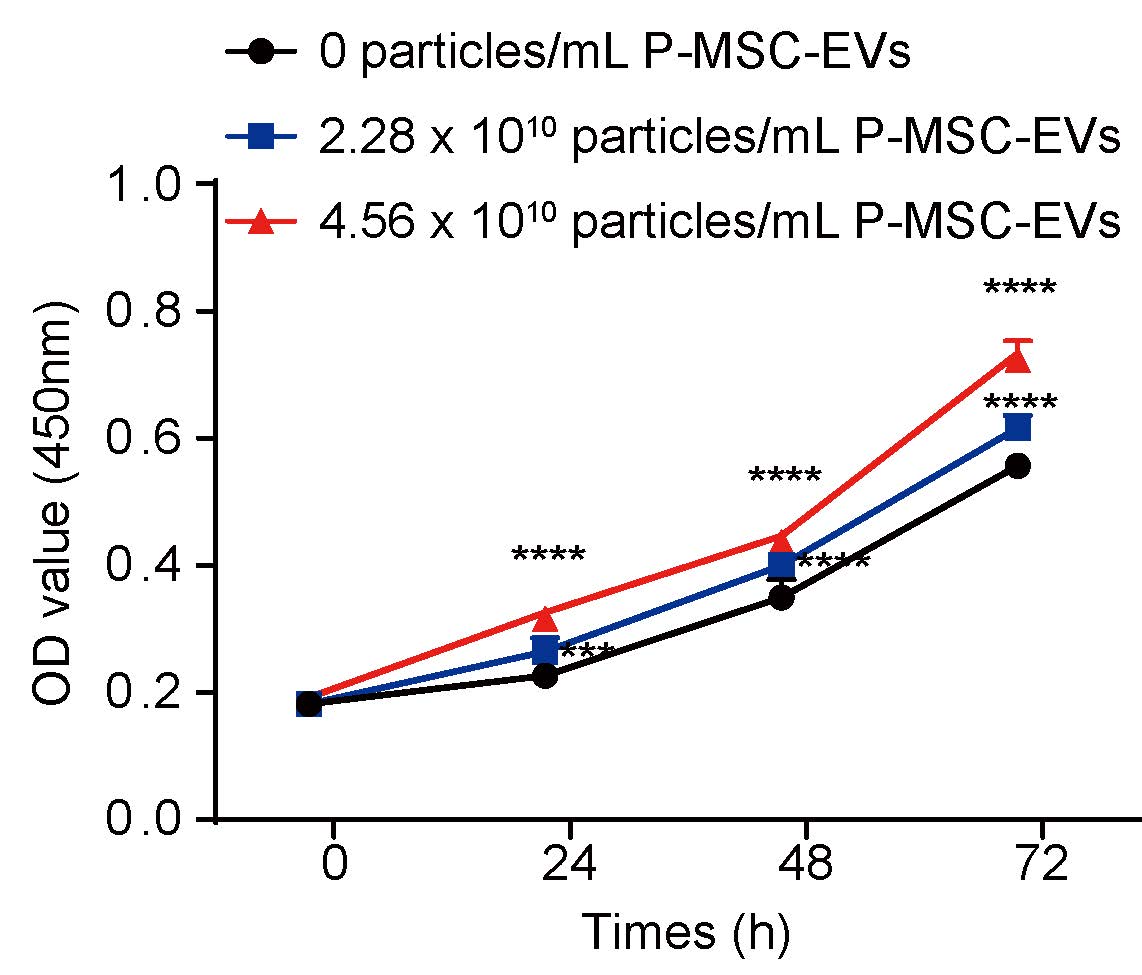
**

**Figure S2. P-MSC-EVs enhanced the proliferation of HG-induced senescent HDFs.** HG-induced HDFs were treated with P-MSC-EVs at different concentrations. At 24 h, 48 h, and 72 h after treatment, the cellular proliferation was measured by CCK-8 assay. n = 4 per group. Compared with the control group. *** *p* < 0.001; **** *p* < 0.0001.

**
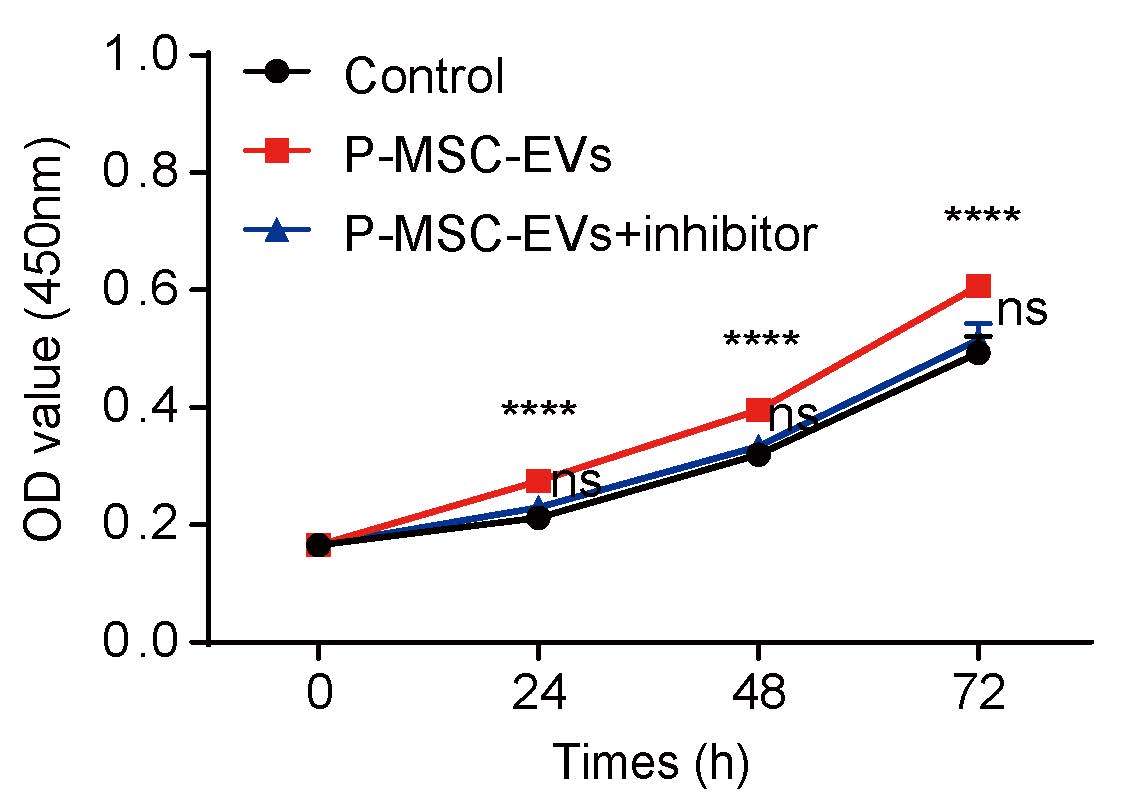
**

**Figure S3. The miR-145-5p inhibitors blocked the effect of P-MSC-EVs on HG-induced senescent HDFs.** The effect of miR-145-5p inhibitors on P-MSC-EVs-induced HDF proliferation was measured by CCK-8 assay. n = 4 per group. Compared with the control group. **** *p* < 0.0001; *ns* no significance.

**
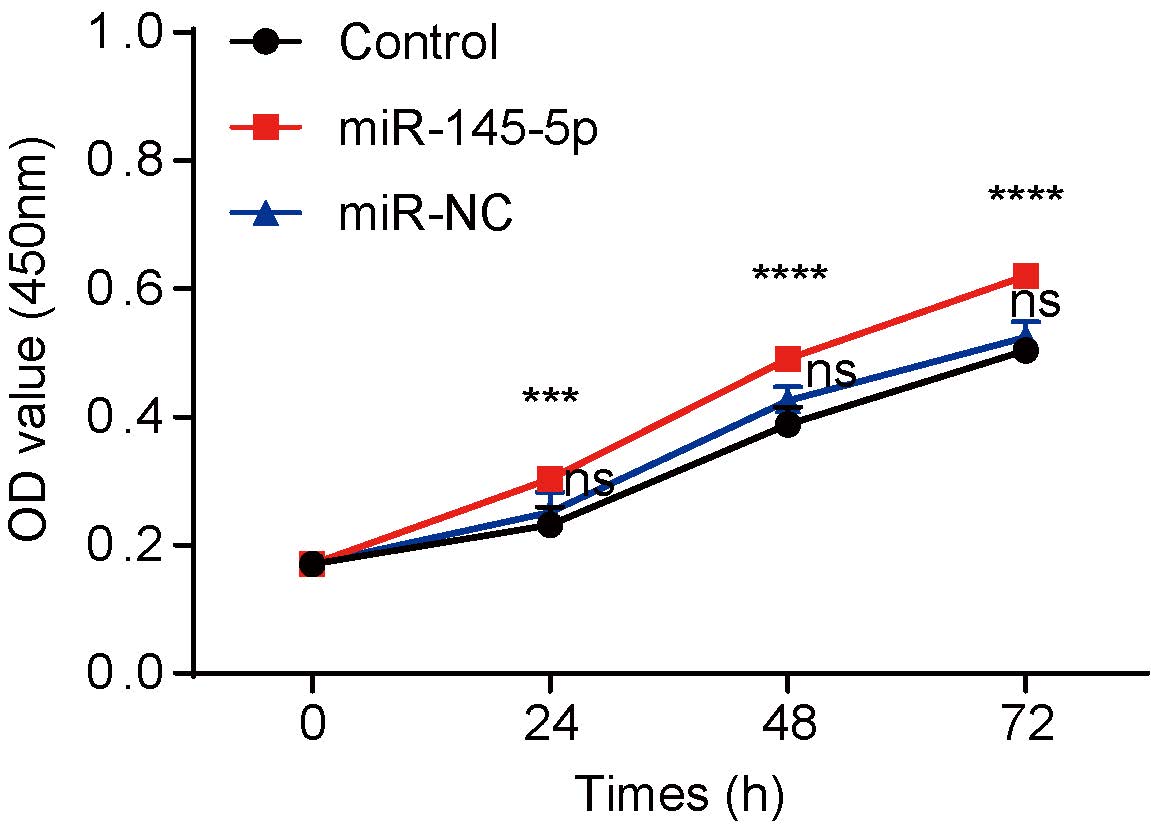
**

**Figure S4. miR-145-5p improved the proliferation of HG-induced senescent HDFs.** Cell proliferation was measured by CCK-8 assay. n = 4 per group. Compared with the control group. *** *p* <0.001; **** *p* < 0.0001. *ns* no significance.**
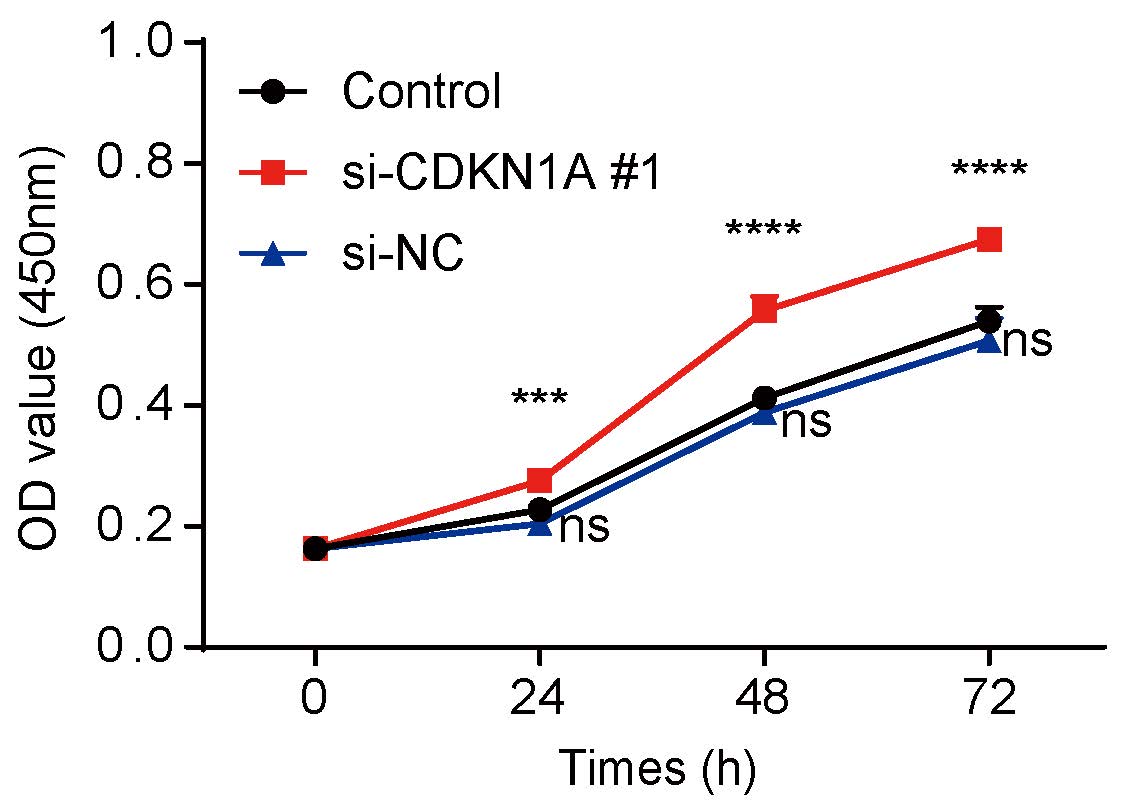
**

**Figure S5. Si-CDKN1A #1 enhanced the proliferation of HG-induced senescent HDFs.** Cell proliferation was measured by CCK-8 assay. n = 4 per group. Compared with the control group. *** *p* < 0.001; **** *p* < 0.0001. *ns* no significance.


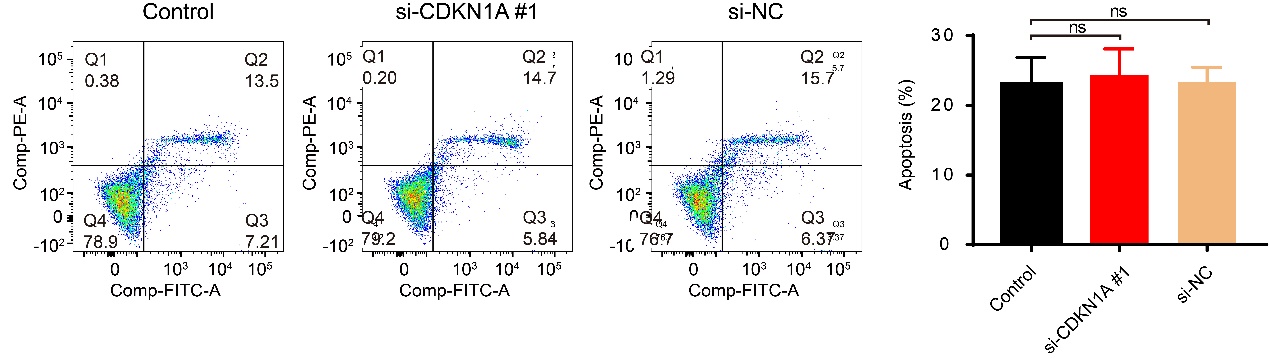


**Figure S6. CDKN1A inhibition had no effect on apoptosis rate of HG-induced senescent HDFs.** Apoptosis rates of HG-induced senescent HDFs were detected by flow cytometry. n = 3 per group. Compared with the control group. *ns* no significance.


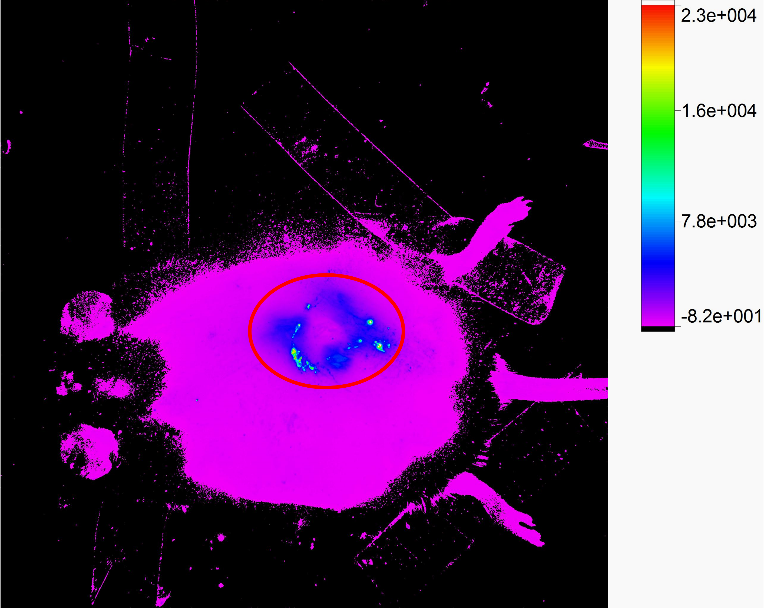


**Figure S7. Retention of agomiR-145-5p in skin tissues.** Representative images of agomiR-145-5p retention in skin tissues on Day 2 after injection. Blue zone in red circle indicates injected agomiR-145-5p labeled with Cy3.
